# Potential Therapeutics Targeting Upstream Regulators and Interactors of EHMT1/2

**DOI:** 10.3390/cancers14122855

**Published:** 2022-06-09

**Authors:** Gareth Chin Khye Ang, Amogh Gupta, Uttam Surana, Shirlyn Xue Ling Yap, Reshma Taneja

**Affiliations:** 1 Healthy Longevity Translational Research Program, Department of Physiology, Yong Loo Lin School of Medicine, National University of Singapore, Singapore 117593, Singapore; a.c.k.gareth@u.nus.edu (G.C.K.A.); e0672667@u.nus.edu (A.G.); 2 Department of Pharmacology, Yong Loo Lin School of Medicine, National University of Singapore, Singapore 117593, Singapore; mcbucs@imcb.a-star.edu.sg; 3 Institute of Molecular and Cell Biology, Agency for Science, Technology and Research A*STAR, 61 Biopolis Drive, Singapore 138673, Singapore; 4 Department of Medicine, Yong Loo Lin School of Medicine, National University of Singapore, Singapore 119228, Singapore; shirlyn_yap@gis.a-star.edu.sg

**Keywords:** lysine methyltransferases, upstream regulators, interactome, post-translational modifications, therapeutics

## Abstract

**Simple Summary:**

The expression of Euchromatin histone lysine methyltransferase 1 and 2 (EHMT1/2) is deregulated in many cancers. Most studies thus far have focused on the downstream targets and pathways regulated by EHMTs. However, the mechanisms that lead to their deregulated expression, and the interacting proteins that could impact EHMT activity are not well understood. In this review, we summarize our current understanding of the upstream regulators and the interactors that provide alternative therapeutic approaches to tackle EHMT driven malignancies.

**Abstract:**

Euchromatin histone lysine methyltransferases (EHMTs) are epigenetic regulators responsible for silencing gene transcription by catalyzing H3K9 dimethylation. Dysregulation of EHMT1/2 has been reported in multiple cancers and is associated with poor clinical outcomes. Although substantial insights have been gleaned into the downstream targets and pathways regulated by EHMT1/2, few studies have uncovered mechanisms responsible for their dysregulated expression. Moreover, EHMT1/2 interacting partners, which can influence their function and, therefore, the expression of target genes, have not been extensively explored. As none of the currently available EHMT inhibitors have made it past clinical trials, understanding upstream regulators and EHMT protein complexes may provide unique insights into novel therapeutic avenues in EHMT-overexpressing cancers. Here, we review our current understanding of the regulators and interacting partners of EHMTs. We also discuss available therapeutic drugs that target the upstream regulators and binding partners of EHMTs and could potentially modulate EHMT function in cancer progression.

## 1. Introduction

Epigenetic regulation refers to mechanisms that influence gene expression without an alteration to the original DNA sequence. This dynamic network involves the cooperative effort and tight regulation of multiple epigenetic factors. Since the precise integration of various epigenetic factors is crucial for the proper function of many biological processes, a deregulation of epigenetic modifications often results in various diseases, such as cancer, autoimmune diseases, and developmental abnormalities [1,2,3]. Besides DNA methylation and chromatin remodeling, histone modification is a main form of epigenetic control. The post-translational modification of histones by methylation, acetylation, ubiquitination, SUMOylation, and phosphorylation can alter chromatin structure and transcription factor accessibility, thereby influencing gene transcription [4].

First described in the early 1960s, methylation is now one of the most well-characterized forms of post-translational histone modifications [5]. Histone methylation mainly happens on the side chain of lysine and arginine residues and is generally associated with transcriptional repression [6]. Lysine-specific methyltransferases (KMTs) are responsible for catalyzing the addition of methyl groups to the lysine residues present at the N-terminal tails of core histones. Growing interest in histone lysine methylation over the past decades has uncovered new roles for KMTs in key biological processes [7]. This review focuses on EHMT1/2 which belong to the Su(var)3-9, enhancer of zeste, and trithorax (SET) domain-containing KMTs. EHMTs are the main methyltransferases responsible for catalyzing mono- and dimethylation on the K9 residue on histone H3 (H3K9me1/2) [8,9]. In addition, they can also function as co-activators or protein scaffolds independently of their methyltransferase activity [10].

EHMT1/2 expression is commonly dysregulated in multiple human malignancies, such as breast, liver, lungs, brain, and ovarian cancers [11,12,13,14,15]. Although EHMT1/2 are viewed mainly as oncogenes, a few studies have shown that they can have tumor-suppressive functions [16,17]. The contrasting roles of EHMTs in cancer may be attributed, in part, to the proteins EHMTs interact with, which then determines the downstream targets of EHMTs to affect tumorigenesis.

EHMTs are attractive therapeutic targets in cancers. However, although several selective inhibitors have been developed to therapeutically target EHMT activity, none of them have made it past clinical trials due to poor physiochemical and pharmacokinetic properties [18,19]. Moreover, these inhibitors will not be effective against the methyltransferase-independent functions of EHMT1/2. Therefore, alternative therapeutic approaches are needed to treat cancers with deregulated EHMT1/2 expression. One way to accomplish this involves targeting the upstream regulators and interacting partners of EHMTs.

## 2. Structure and Function of EHMTs

EHMT1 (GLP) and EHMT2 (G9a) are closely related enzymes that catalyze the transfer of methyl groups from the substrate S-adenosyl methionine (SAM) to target amino acids. As lysine methyltransferases, they catalyze methylation on the lysine residues of histone tails, specifically histone 3 lysine residue 9 (H3K9), where they predominantly deposit either two (H3K9me2) or three (H3K9me3) methyl groups [20]. These marks are then recognized by chromatin remodeling complexes aiding the formation of a condensed chromatin state. The H3K9me2/H3K9me3 marks around the promoter region also hinder transcriptional machinery access, thus silencing gene expression [15,20].

EHMT2 and EHMT1 share almost 80% of their amino acid sequences and, thus, various domains [21]. The catalytic activity of EHMTs is mediated by the SET domain, an evolutionarily conserved 130–140 amino acid motif. Along with the pre-SET and post-SET domains, this motif is responsible for SAM binding and subsequent methyltransferase activity [22]. The L-shaped core of the SET domain binds SAM to a lysine residue of the target. G9a and GLP also recognize methylated lysine residues by means of ankyrin (Ank) repeats adjacent to the SET domain [23]. The 33-amino-acid residue binds to methylated lysine residues at the N terminal of H3 [24]. As a result, EHMTs function as both epigenetic writers and readers through their methyltransferase and recognition properties.

In addition to the methylation of histones, EHMTs form complexes with and methylate other protein targets, such as p53 (K372), Wiz (K305), CDYL1 (K135), ACINUS (K654), and MyoD (K104) [25,26]. The Ank repeats are responsible for these interactions and, in turn, impact the functional/transcriptional activity of the interacting partner [25].

## 3. EHMT1/GLP and EHMT2/G9a Dysregulation in Cancer

As EHMTs lie upstream of various targets and pathways, their dysregulated expression is associated with many diseases, especially cancer. The expression of EHMT2 and EHMT1 is upregulated in various cancers and is correlated with poor clinical outcomes [15].

G9a is frequently overexpressed in hepatocellular carcinoma (HCC) and silences the tumor suppressor phospholipase A and acyltransferase 4 (RARRES3) [27]. Likewise, G9a is upregulated in melanoma patients, with its expression correlating to poor disease outcomes [28]. IHC studies in 107 pairs of gastric cancer samples showed increased nuclear staining compared to matched normal tissues [29]. The levels of G9a were also significantly higher in metastatic samples than in primary tumor samples, and this was associated with complex formation with lysine acetyltransferase 2B (P300) and the glucocorticoid receptor (GR), resulting in the increased expression of integrin subunit beta 3 (ITGB3) and promoting peritoneal metastasis [29]. The increased expression of G9a in breast cancer and head and neck squamous cell carcinoma was also shown to repress E-cadherin, thus promoting metastasis [30,31]. In endometrial cancer, the upregulation of G9a was associated with myometrial invasion through the silencing of E-cadherin [32]. Lung adenocarcinoma patients with higher expression of G9a had poorer prognosis. Moreover, significantly higher levels of G9a were observed in tumor-initiating cells isolated from non-small-cell lung cancer (NSCLC) patients [33,34].

Although less widely analyzed, GLP/EHMT1 overexpression also correlates with poor prognosis in various cancers where its depletion led to positive outcomes. In esophageal squamous cell carcinoma (ESCC), GLP expression was shown to be significantly higher in preinvasive lesions compared to paired normal tissues [35]. A significant difference in survival was also observed when cohorts were separated according to GLP expression [35]. Similarly, GLP is upregulated in lung and gastric cancers [36,37]. The expression of GLP in gastric cancer was shown to promote tumor progression though E-cadherin silencing [37]. The upregulation of GLP was also observed in rhabdomyosarcoma, where its depletion led to the decreased motility and increased differentiation of cancer cells [38].

Thus, both G9a and GLP are dysregulated in cancer and influence the expression of various downstream targets to promote proliferation, migration, and metastasis associated with poorer prognosis in patients [39]. Epigenome reprogramming occurs during the transition from normal to tumorigenic and metastatic states, resulting in altered methylation patterns. Chromatin changes during differentiation result in the formation of large organized chromatin K9 modifications (LOCKs) that are largely dependent on H3K9me2 marks [40]. LOCKs are lost during EMT and in cancer cell lines [41,42,43]. A reduction in heterochromatic H3K9me2 mark and an increase in euchromatin marks H3K4me3 and H3K36me3 are seen during EMT that are localized to LOCKs. DNA hypomethylation in regions of LOCKs was also observed, resulting in a high expression of cell cycle genes.

## 4. Pharmacological Inhibitors of EHMTs and Their Limitations

As EHMTs are dysregulated in various cancers, selective inhibitors have been developed to therapeutically target them [44]. Because the oncogenic roles of G9a have been better characterized compared to GLP, many of the inhibitors developed thus far target G9a activity. Nonetheless, these inhibitors also inhibit GLP at a higher dosage. EHMT inhibitors can be classified into two categories: SAM-competitive inhibitors and substrate-competitive inhibitors [18]. SAM-competitive inhibitors such as BRD9539, BRD4770, and CBC-12 compete with SAM, thereby interfering with EHMT methyltransferase activity [45,46]. On the other hand, substrate-competitive inhibitors bind to the histone binding pocket of EHMTs. BIX01294 was the first EHMT substrate-competitive inhibitor and served as a template for designing subsequent substrate-competitive inhibitors such as UNC00224, UNC0321, UNC0638, and UNC0642 [47,48,49,50].

Although EHMT inhibitors are effective in pre-clinical models, they have not progressed to clinical use due to poor lipophilicity and pharmacokinetics, a lack of specificity, and high toxicity [18,19,44]. Another limitation is that current inhibitors target only EHMT methyltransferase activity. Although inhibiting methyltransferase activity is, to a large extent, able to inhibit the major function of EHMTs, it is unable to target their methyltransferase-activity-independent functions [51].

Therefore, there is a need to explore alternative avenues to target EHMTs in the hope of treating malignancies with EHMT dysregulation. In this we review, we summarize the upstream regulators of EHMT expression and their known interacting partners. As these factors represent potential approaches to indirectly target EHMTs, we also evaluate the potential of current drugs against these upstream regulators or interactors to mitigate EHMT-driven or EHMT-complex-driven cancers.

## 5. Upstream Regulators of EHMT1/2

**Figure 1:**
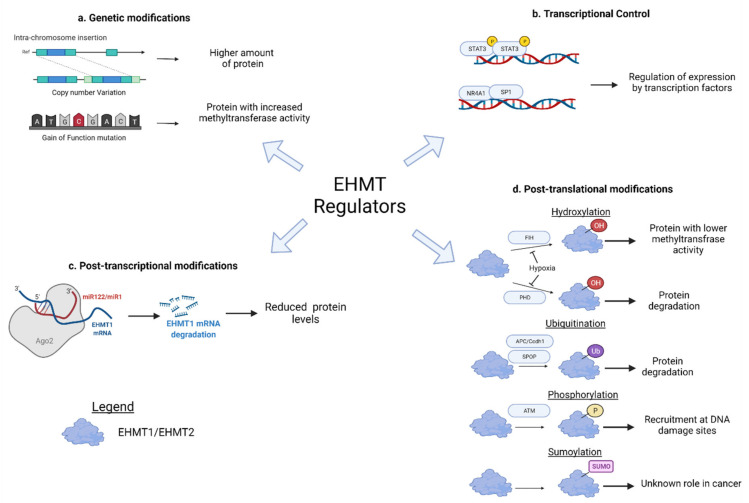
Mechanisms leading to dysregulation of EHMTs. (a) Copy number alterations and mutations in EHMT lead to increased expression. (**b**) Transcription factors that bind to the upstream regulatory elements in the EHMT promoter, leading to its expression. (**c**) Specific miRNAs that target and degrade EHMT mRNAs. (**d**) EHMT stability and function are affected by various post-translational modifications.

Mutation and copy number alterations, transcriptional regulation, and post-transcriptional and post-translational regulation (Figure 1 and Table 1) lead to EHMT dysregulation.

### 5.1. Mutation and Copy Number Alterations

Gain-of-function point mutations were found on the glycine 1069 residue located at the SET methyltransferase domain of G9a [52]. This point mutation changes glycine to either leucine or tryptophan and increases the catalytic activity of G9a in melanoma cells. EHMT copy number variation (CNV) is also commonly seen in cancer. CNV refers to a variation in the copy number of genes in a chromosomal segment between individuals. In melanoma, HCC, and CRC, copy number gains were identified at the 6p21 locus (chr6: 30,950,307–33,085,850), which harbors G9a, leading to G9a overexpression [27,52,57]. However, the mechanism responsible for the 6p21 locus copy number gain is unclear. Similarly, a GLP copy number gain was also observed in breast cancer, resulting in an increase in GLP expression [76].

### 5.2. Transcriptional Regulation

Epidermal growth factor receptor (EGFR) signaling has been shown to positively regulate G9a expression through signal transducer and activator of transcription 3 (STAT3) in EGFR^+^ lung cancer [58]. Treatment with BB1608, a STAT3 inhibitor, results in decreased G9a and HER3 expression and sensitizes lung cancer cells to EGFR tyrosine kinase inhibitor [58].

As opposed to STAT3, the special AT-rich sequence binding protein 2 (SATB2) transcription factor suppresses G9a expression and mitigates the invasiveness of lung cancer [77]. In alveolar rhabdomyosarcoma (ARMS), the orphan nuclear receptor 4A1 (NR4A1) is responsible for G9a overexpression by complexing with the Sp1 transcription factor (Sp1) and occupying the -511 GC-rich region of the G9a promoter [61].

### 5.3. Post-Transcriptional Regulation

EHMT expression can also be affected by post-transcriptional regulation. The expression of miR-122, a tumor suppressor, correlates inversely with G9a levels in hepatocellular carcinoma (HCC) [64]. miR-122 specifically targets the mRNA of G9a. The overexpression of miR-122 in HCC attenuates G9a expression, thus abrogating colony formation and the invasiveness of HCC [64]. miR-1 was also reported to be a negative regulator of G9a and is often downregulated in HCC [27].

### 5.4. Post-Translational Regulation

The effect of PTMs on EHMT activity and stability remain largely unexplored. EHMTs undergo auto-methylation at the K239 residue [78]. While auto-methylation does not affect the stability or activity of EHMTs, it enhances the interaction with the heterochromatin protein 1 γ (HP1γ) [78]. The EHMT-HP1γ complex functions as an activator, increasing GR target genes to enhance leukemia cell death [79].

EHMTs are also subjected to hydroxylation by the oxygen sensor asparaginyl hydroxylase factor inhibiting HIF (FIH) [66]. Under normoxic conditions, FIH hydroxylates GLP at Asn867 and G9a at Asn779, thereby repressing their activities [66]. G9a/GLP escape FIH-mediated hydroxylation in hypoxic conditions and repress metastasis suppressor genes, thereby inducing metastasis in ovarian cancer [66]. Breast cancer studies showed that G9a can be hydroxylated on proline residues 676, 1194, and 1207 by prolyl hydroxylase domain 1 (PHD1) [69]. Proline hydroxylation is important for the effective proteasomal degradation of G9a. Under hypoxic conditions, proline hydroxylation on G9a is impaired, thereby stabilizing G9a and increasing G9a’s repressive activity [69].

EHMT1/2 stability is also dependent on ubiquitination. Speckled-type POZ protein (SPOP), an E3 ubiquitin-protein ligase, has been reported to promote the ubiquitination and proteasomal degradation of EHMTs [70]. In a study on prostate cancer patients, SPOP was subjected to hemizygous missense mutation. Mutant SPOP antagonized the functions of wild-type SPOP, thereby reducing the SPOP-mediated degradation of EHMTs [70]. This drives the EHMT-mediated silencing of tumor suppressor genes such as forkhead Box O1 (FOXO1), GATA binding protein 5 (GATA5), and N-myc downstream regulated 1 (NDDRG1) in prostate cancer [70].

Senescence is mostly regarded as a tumor-suppressive process by repressing cancer cell proliferation and malignant transformation [80]. In senescent cells, both G9a and GLP are ubiquitinated by the APC/C^cdh1^ ubiquitin ligase [81]. This results in the proteasomal degradation of G9a and GLP, causing a decrease in H3K9 dimethylation marks globally and on the promoter of interleukin 6 and 8 (*IL-6* and *-8*) [81], inducing the expression of *IL-6* and *8,* which are important in enabling the senescence-associated secretory phenotype [74].

G9a can be phosphorylated by the ATM serine/threonine kinase (ATM) on serine 569 residue, which is required for its recruitment to DNA break sites on the chromatin [75]. The presence of G9a further recruits p53 binding protein 1 (53BP1) and BRCA1 to the break site, initiating DNA repair [75]. This causes osteosarcoma cells to be resistant to ionizing radiation. In addition to the aforementioned PTMs, EHMTs are also subjected to SUMOylation. However, its role in cancer is yet to be determined [21,82]. EHMT upstream regulators are summarized in Table 1.

## 6. Interacting Partners of EHMT1/2

**Figure 2:**
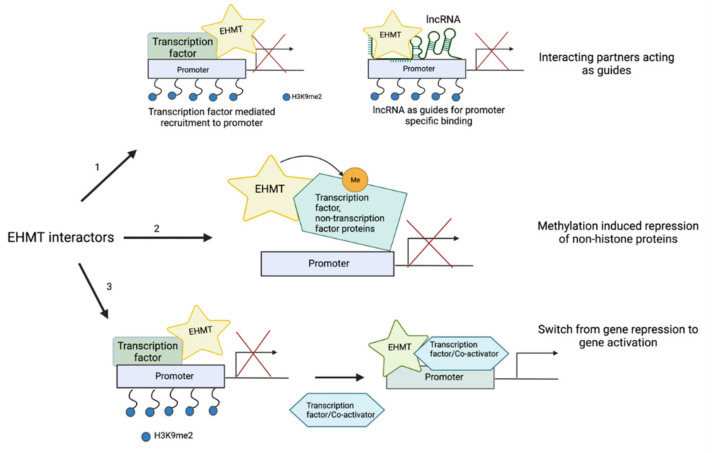
Impact of EHMT-interacting proteins. (1) Transcription factors and lncRNA can bind to EHMTs and guide them to the promoter of target genes to suppress gene transcription. (2) EHMTs can methylate their binding partners, rendering them functionally inactive. (3) Upon binding to specific interactors, EHMTs can function to activate transcription.

Because EHMTs lack a DNA-binding domain, the associations with various transcription factors/co-factors, zinc-finger containing proteins, epigenetic regulators, and, in some cases, non-coding RNA are essential for its function [83]. These interactors act either as guides for EHMTs to specific chromatin sites or they interact with EHMTs to change the overall function of the complex to activate gene expression (Figure 2 and Table 2) [51]. For most cases, EHMTs’ role as an activator is independent of their methyltransferase activity; instead it is dependent on the interactors with which they associate [84,85]. Some of the interacting partners are also methylated by EHMTs, indicating that they could also function as downstream substrates. This adds a layer of complexity to the regulation of their target genes, highlighting the need for a further understanding of EHMT complexes in different cancers.

**Table 2:** EHMT-interacting proteins in distinct cancer types. The roles and potential therapeutics are summarized.

Interactors	G9a/GLP	Function	Cancer Type	Phenotype	Potential Therapeutics
**Transcription factors**					
MDM2	GLP	Cancer	Osteosarcoma [86]	Avoid p53-induced cell death	Nutlin analogs [87] MI-219 [88]
P53	GLP and G9a	Cancer	CRC [89] HCC [17]	Cell cycle progression Escaping apoptosis	Nutlin analogs [87] MI-219 [88]
			Lung cancer (activator) [90]	Enhance apoptosis and reduce colony formation	Nutlin analogs [87] MI-219 [88] KJ-pyr-9 [91] Omomyc [92]
MYC	G9a	Cancer	Breast cancer [93,94]	Cell proliferation	
STAT3	G9a	Cancer	GC [95] Breast cancer [96]	Evading autophagy EMT and CSC maintenance	SH003 [97] STA-21 [98] Stattic [99] IS3295 [100] Cisplatin [100]
FOXO1	G9a	Cancer	CRC [101]	Cell proliferation	Troglitazone [102] Gallic acid [103] Skp2E3LIs [104] NSC689857 [105] Linichlorin A [106]
RUNX3	G9a	Cancer	GC [107]	Cell proliferation suppresses apoptosis and immune response	-
RUNX2	G9a	Cancer	Breast cancer [108] Prostate cancer [108]	Metastasis	-
TBX2	G9a	Cancer	Breast cancer [109]	Cell proliferation	-
NKX3.1	G9a	Cancer	Prostate cancer [110]	Inhibit cell differentiation	-
**Zinc finger proteins**					
WIZ	G9a and GLP	Maintenance of pluripotency	-	-	-
Snail	G9a	Cancer	Breast cancer [111]	EMT Cell proliferation Metabolic reprogramming CSC maintenance	SD-093 [112] LY2157299 [113] AP12009 [114] ISTH0036
Slug	G9a	Cancer	HCC [115] Lung cancer [115]	EMT	SD-093 [112] LY2157299 [113] AP12009 [114] ISTH0036 [116]
ZNF644	G9a	Neurodevelopment, maintenance of pluripotency	-	-	-
ZNF518B	G9a	Cancer	CRC [117]	Cell proliferation	-
**Non-transcription factor proteins**					
Cyclin D	G9a	Cancer	Breast cancer [118]	Cell proliferation	-
RPA	G9a	Cancer	CRC [119]	Radio and chemoresistance	-
MT1h	GLP	Cancer	HCC [120] Prostate cancer [120]	Reduce cell cycle Reduce Migration and invasion Reduce colony formation	-
**Epigenetic regulators**					
EZH2	G9a	Cancer	Breast cancer [121]	Cell proliferation	GSK343 [121] GSK2816126 [122]
	GLP	Repressive complex	-	-	-
HDACs	G9a	Cancer	HCC [115]	EMT Migration and invasion	TSA [123]
DNMTs	G9a	Cancer	Hematological malignancies [124]	Cell proliferation Inhibit apoptosis	CM-272 [124] Azacytidine [71] Decitabine [72]
CDYL	G9a and GLP	Cancer	Osteosarcoma [125] HCC [126]	Cell proliferation	D03 [127]
CDYL2	G9a and GLP	Cancer	Breast cancer [128]	Migration Sphere formation	-
**Long non-coding RNAs**					
TERNA1	G9a	Cancer	HCC [129] Osteosarcoma [129]	EMT Migration and invasion	-
NEAT1	G9a	Cancer	HCC [130]	EMT Migration and invasion	-
HOTAIRM1	G9a	Cancer	Osteosarcoma [131] GBM [131]	Cell proliferation Migration and invasion Reduce apoptosis	-

### 6.1. Transcription Factors

#### 6.1.1. P53

EHMTs interact with p53 to regulate cancer cell proliferation and apoptosis [17,89,132]. This interaction leads to the inactivation of p53 by the methylation of the lysine residue K373 [133]. Mouse double minute 2 homolog (MDM2), an E3 ubiquitin ligase, binds to p53 and augments its inactivation in an EHMT-dependent manner [86]. The EHMT-dependent inactivation of p53 also increases the expression of polo-like kinase 1 (PLK1), a serine/threonine kinase that phosphorylates and activates essential cell cycle regulators such as cyclin B and CDC25C (cell division cycle 25C) [89]. The increase in PLK1 expression promotes cell growth and proliferation in colorectal cancer. EHMTs are also recruited to the cyclin-dependent kinase inhibitor 1A (*p21*) promoter through the acidic domain of MDM2, resulting in H3K9 methylation and the repression of its transcription [86].

Besides the methylation of p53, G9a also competes with p53 for binding to the promoter region of BCL-like 14 (*Bcl-G*), a pro-apoptotic gene of the BCL-2 family [134]. The binding of G9a to the promoter silences Bcl-G gene expression, contributing to tumor initiation in hepatocellular carcinoma (HCC). Interestingly, a recent study showed that EHMTs can promote p53-dependent apoptosis. p53 localization at the promoter of the BH3 pro-apoptotic gene Puma induces its transcription [17]. G9a was shown to be essential for the localization of p53 and histone acetyltransferase (HAT) CBP/p300 to the Puma promoter, driving its expression and enhancing caspase-mediated apoptosis in lung cancer [90]. However, the mechanism by which G9a influences p53 recruitment has not been elucidated.

#### 6.1.2. MYC

The MYC oncogene is deregulated in multiple cancers, and its overexpression increases cyclins (A and E) and CDK expression [135]. EHMTs interact with MYC through the MYC box II domain [93]. This interaction is abolished with a mutant MYC lacking the MYC box II domain. The G9a-MYC complex in breast cancer localizes at the *p21* and *GADD45A* promoters to catalyze H3K9 methylation, repressing both MYC target genes [93,94]. In addition to interacting with MYC in glioblastoma, G9a positively regulates *MYC* transcription in a methyltransferase-independent manner by occupying the −2267 to −1949 region on the *MYC* promoter [136]. The upregulation of MYC increases cell proliferation, migration, invasion, and clonogenicity of glioblastoma cells. The knockdown of G9a thus dampens the oncogenic role of MYC, thereby inhibiting tumor growth in vitro and in vivo.

#### 6.1.3. STAT3

The signal transducers and activators of transcription (STATs) encompass a group of cytoplasmic transcription factors that, upon activation by phosphorylation, participate in the transmission of signals from cell surface receptors to the nucleus [137]. Upon phosphorylation by Janus kinases (JAK) on a conserved tyrosine residue, STATs dimerize and translocate into the nucleus to transactivate target genes. While in most cases STAT proteins primarily function as transcriptional activators, some studies have shown that they can cause transcriptional repression by recruiting repressive cofactors [138].

Activated STAT3 has been shown to interact with G9a to form a repressive complex. In hypoxic conditions, the STAT3-G9a complex inhibits autophagy in gastric cancer (GC) [95]. The treatment of cells with SH003, an herbal formulation, induces autophagy by abrogating the interaction between STAT3 and G9a [95]. The disruption of the STAT3-G9a complex causes activating transcription factor 4 (ATF4) to displace G9a and occupy the promoter of microtubule associated protein 1A/1B light chain 3B (*MAP1LC3*), increasing the expression of *MAP1LC3B* and promoting autophagy [95]. The knockdown of either STAT3 or G9a, or treatment with a G9a inhibitor, BIX-01294, yielded similar outcomes as SH003 treatment, suggesting that the STAT3-G9a complex enables GC cells to evade autophagy.

The STAT3-G9a complex has also been reported to epigenetically silence the expression of miR-200c by depositing H3K9me2 marks on its promoter in MCF12A breast cancer cells [96]. Silencing miR-200c de-represses miR-200 target genes such as zinc finger E-box binding homeobox1 (*ZEB1*) and bombyx mori nucleopolyhedrovirus (*BM11*) and promotes EMT and CSC formation [96]. A pharmacological inhibition with the STAT3 inhibitor S3I-201 was shown to block the STAT3-G9a interaction and decrease H3K9me2 marks on the miR-200c promoter, thereby inhibiting EMT, inducing autophagy, and reducing the CSC population in breast cancer. This highlights the importance of the interaction between STAT3 and G9a in driving tumorigenesis.

#### 6.1.4. Other Transcription Factors

Various studies have shown that FOXO1 has a tumor-suppressive role and plays a part in regulating cell proliferation and apoptosis. FOXO1 activity is regulated by the PI3K/AKT signaling pathway through the phosphorylation of FOXO1 by AKT [139]. The phosphorylation of FOXO1 inhibits FOXO1-dependent transcription by impairing FOXO1’s DNA-binding capability and increasing FOXO1’s binding affinity to 14–3–3 protein, resulting in the expulsion of the FOXO1–14–3–3 complex from the nucleus [140]. G9a affects the stability of FOXO1 in a methylation-dependent manner [101]. G9a interacts with and methylates FOXO1 at K273. This methylation enhances the interaction of FOXO1 with S-phase kinase-associated protein 2 (SKP2), an E3 ubiquitin ligase, resulting in the degradation of FOXO1 and increasing colon cancer cell proliferation [101]. Additionally, tissue samples of human colon cancer showed an inverse correlation between G9a and FOXO1 levels, with lower FOXO1 expression correlating with a poorer prognosis [101].

Similarly, RUNX family transcription factor 3 (RUNX3) can be methylated by G9a at K129 and 171, suppressing RUNX3 transactivation activity [107,141]. As observed in GC during hypoxia, the methylation of RUNX3 prevents its interaction with core-binding factor subunit beta (CBFβ) and histone acetyltransferases P300 (P300) [107]. This impairs the binding of the complex to promoters and hinders the transactivation of target genes that regulate cell proliferation and apoptosis. The expression of the K129R and K171R RUNX3 mutants inhibits methylation by G9a, thereby decreasing the expression of genes involved in proliferation (PI3KC, PLK4, and SMC4) while increasing those related to apoptosis (TRIM22 and BCL2L1) and the immune response (NLRP3). RUNX2 also functions as a chaperone for the recruitment of G9a to endogenous RUNX2 binding sites on the chromatin, activating RUNX2 target genes MMP9, CST7, SDF1, and CSF, which are known to drive EMT and metastasis in breast and prostate cancers [108].

The breast cancer oncogene T-box transcription factor 2 (TBX2) forms a complex with G9a and polycomb repressor complex 2 (PRC2) through the T-Box domain of TBX2 [142]. TBX2-G9a-PRC2 catalyzes the H3K9me2/3 methylation of histone H3 on the promoter of N-Myc downstream-regulated gene 1 protein (*NDGR1*), suppressing its expression and increasing the cell proliferation of breast cancer cells [109]. Treatment with BIX-01294 reduces cell proliferation, and this is observed to an even greater degree upon combination with DN-TBX2, a mutant TBX2 protein containing an absent T-box domain, suggesting that G9a and TBX2 may have a synergistic role in driving breast cancer cell proliferation [142]. In rhabdomyosarcoma, TBX2 is overexpressed and induces the PI3K/AKT signaling pathway by recruiting HDAC1 to deacetylate the PTEN promoter. Notably, the TBX2-G9a complex may be involved in the silencing of PTEN, as G9a is also overexpressed in RMS [143].

EHMT also functions as a co-regulator for the homeobox-containing transcription factor (NKX3.1) through the homeodomain to activate the transcription of ubiquitously transcribed tetratricopeptide repeats containing Y-linked (*UTY*), mediating prostate cancer cell differentiation; the lack of the homeodomain promotes tumorigenesis [110]. Therefore, in addition to their canonical roles in epigenetic silencing, EHMTs interact with transcription factors to regulate gene expression in a methyltransferase-activity-independent manner.

### 6.2. Zinc Finger Proteins

The Snail family transcriptional repressors 1 (SNAI1) and 2 (SLUG) induce EMT and maintain CSC populations in multiple cancers [144,145]. The SNAI family contains 4–6 C_2_H_2_-type zinc fingers for DNA binding, a central serine-rich domain, and an N-terminal SNAG domain, which acts as a docking site for the binding of corepressors and epigenetic regulators [146]. G9a interacts with Snail via its ankyrin repeats and SET domain, as observed in breast cancer and head and neck squamous cell carcinoma [31]. Together with DNMT1, the G9a-Snail-DNMT1 complex is responsible for the Snail-mediated induction of EMT by binding to the promoter and silencing the expression of E-cadherin by DNA methylation and H3K9 methylation [111].

The G9a-Snail-DNMT1 complex also silences fructose bisphosphatase 1 (FBP1) expression in basal-like breast cancer [147]. The silencing of FBP1 reprograms the cell metabolically by inducing glycolysis, glucose uptake, and ultimately, cell proliferation [148]. A decrease in FBP1 expression also promotes interactions between β-catenin and T-cell factor, resulting in an increase in the CSC-like characteristics of breast cancer cells [149]. As such, the G9a-Snail-DNMT complex induces the β-catenin pathway responsible for the expression of EMT target genes such as fibronectin, vimentin, and α-SMA [149]. In both cases, knocking down either G9a or Snail reinstates the expression of E-cadherin and FBP1, decreasing EMT and CSC maintenance, respectively.

Another member of the SNAI family, Slug, forms a complex with G9a and histone deacetylase 1, 2, and 3 (HDAC1, 2, and 3) in HCC and lung cancer [115]. The G9a-Slug-HDAC1/2/3 complex suppresses the expression of E-cadherin through the deacetylation of H3K4/56 and the methylation of H3K9 at its promoter [150]. Treatment with BIX-01294 or the HDAC inhibitor TSA abolishes the silencing of E-cadherin and reduces EMT in liver and lung cancers.

The ability to guide G9a to specific gene promoters is also observed with other zinc finger transcription factors. ZNF518B drives cell proliferation in CRC by silencing key tumor suppressors such as Peptidyl arginine deiminase 3 (*PADI3*) and Regulator of G protein signaling (*RGS4*) [117]. ZNF518B recruits and directs G9a to the promoters of *PADI3* and *RGS4* and represses their expression by catalyzing H3K9me2 [117]. The knockdown of ZNF518B inhibits G9a occupancy on promoters, restoring the expression of the target genes to decrease cell proliferation and increase apoptosis [117].

Furthermore, two well-known EHMT interactors, WIZ and ZNF644, associate with the N-terminal transactivating domain to direct the complex to the promoter region of pluripotent state maintenance genes cell wall biogenesis 43 C-terminal homolog (*CWH43),* Rho associated coiled-coil containing protein kinase 1 (*ROCK1),* and disco-interacting protein 2 homolog C (*DIP2C*) [151]. A similar observation was made with ZNF281 in embryonic stem cell (ESC) differentiation, wherein the ZNF281-GLP complex is essential for ESC to exit self-renewal and begin differentiation [152].

### 6.3. Non-Transcription Factor Protein

Cyclins are important cell cycle regulators; their activity and expression are tightly controlled to ensure cell cycle progression. Cyclin D1 is important in initiating cell cycle progression, as it results in the phosphorylation of retinoblastoma (RB) through the activation of CDK4 and 6 [153]. The activation of CDK4 and 6 promotes tumorigenesis by antagonizing cytostatic TGF-β signaling and the anti-proliferative transcriptional response through the multisite phosphorylation of SMAD2 and 3 [154]. This results in a switch from a cytostatic to a pro-tumorigenic phenotype. Aside from inducing CDK4 and 6 activity, cyclin D1 binds to G9a through the HTH domain and functions as a chaperone for G9a to the target genes pituitary tumor transforming gene (*Pttg*) and *MDM4*, the regulator of P53 [118]. The knockdown of cyclin D1 abolishes both G9a and H3K9me2 occupancy on the promoter of these genes. Cyclin D1 also ensures the maintenance of G9a-mediated H3K9me2 marks, which are essential in influencing the interaction between nuclear lamina (NL) and the lamina-associated domain (LAD) [118]. The LAD-NL interaction alters chromatin architecture to a more heterochromatin state, repressing genes in these regions [155]. Cyclin D1 and G9a have been shown to be overexpressed in ERα^+^ breast cancer, suggesting that cyclin D1 and G9a may have synergistic roles [118].

Complexes including G9a are also involved in repairing DNA double-strand breaks (DSBs) [75]. DSB repair occurs via homologous recombination (HR) or non-homologous end joining (NHEJ) and is a common pathway that enables cancer cell resistance to chemotherapy or radiotherapy [156]. G9a promotes HR following DSB by first undergoing phosphorylation by casein kinase 2 (CK2) at the Ser211 residue [119]. G9a phosphorylation results in its enrichment at the chromatin regions with DSB where the chromatin-bound G9a interacts with and recruits replication protein A (RPA) [119]. RPA is a heterotrimeric single-stranded DNA binding protein required for efficient HR [157]. The knockdown of G9a reduces the RPA’s chromatin recruitment, foci formation, and the efficacy of HR in CRC, thereby increasing susceptibility to ionizing radiation [119]. Additionally, a decrease in G9a results in fewer cells in the G2/M phase, suggesting that G9a has a role in activating the G2/M DNA damage checkpoint. However, the exact mechanism has not been determined [119].

GLP interacts with metallothionein 1h (MT1h) through the amino acid residue 2–19 on MT1h [158]. MT1h is a tumor suppressor protein that belongs to a class of metal binding proteins that is downregulated in human malignancies such as liver and prostate cancers [120]. MT1h antagonizes Wnt/β-catenin signaling by inhibiting the phosphorylation of Akt and, hence, the phosphorylation of GSK-3β [159]. This enables GSK-3β to phosphorylate and destabilize β-catenin and prevents β-catenin translocation to target genes. As observed in HCC and prostate cancer, the formation of the MT1h-GLP complex is critical for the MT1h tumor-suppressive effect, as a mutation in MT1h inhibits GLP binding and abolishes the tumor-suppressive activity of MT1h [158].

### 6.4. Epigenetic Regulators

EHMTs have also been shown to form functional complexes with other epigenetic regulators such as suppressor of variegation 3–9 homolog 1 (SUV39H1), SET domain bifurcated histone lysine methyltransferase 1 (SETDB1), enhancer of zeste 2 polycomb repressive complex 2 subunit (EZH2), HDACs, and DNMTs to carry out transcriptional repression [160,161,162,163]. Although several reports have highlighted that combinatorial treatment with both inhibitors of EHMT and other regulators entails better efficacy compared to that of a single inhibitor, the importance of the EHMT associations with other epigenetic regulators in the context of cancer are just starting to be explored. In breast cancer, the combined inhibition of G9a and EZH2 by UNC0642 and GSK343, respectively, had a greater effect on gene transcription, inhibiting cancer cell proliferation to a larger extent compared to either drug alone [121]. The dual inhibition of G9a and EZH2 with UNC0637 and GSK2816126 also exerted strong anti-tumor effects in multiple myeloma cells [122]. In hematological malignancies, the dual inhibition of G9a and DNMTs with CM-272, a newly discovered small molecule compound, inhibited proliferation and promoted immunogenic cell death and apoptosis [124].

#### CDYL

The chromodomain Y-like protein (CDYL) is a well-known interactor of EHMTs [164]. CDYL contains an N-terminal chromodomain that recognizes and binds to H3K9me2/3 and H3K27me3 [165]. It also contains a C-terminal CoA pocket that allows its function as a corepressor by reducing histone lysine crotonylation marks on promoters of genes such as Ras homolog family member A (RhoA), brain-derived neurotrophic factor (BDNF), sodium voltage-gated channel alpha subunit 8 (SCN8A), VGF nerve growth factor inducible (VGF), and E-cadherin [125,166].

In osteosarcoma, CDYL is critical in the preservation of the epigenetic landscape from parent to offspring cells, as it recruits EHMTs to replication forks during S phase and represses H3K9me2/3 marks deposited on newly synthesized histone H3 [125]. The knockdown of CDYL halts early S phase progression and increases the susceptibility of cells to DNA damage. The interaction between G9a and CDYL is crucial in driving hepatocellular carcinoma (HCC) tumor progression [126]. Cells with high G9a and CDYL expression showed more intense Ki-67 and survivin staining, suggesting that G9a and CDYL are involved in HCC proliferation and apoptosis inhibition [126].

CDYL2, the homolog of CDYL, co-immunoprecipitates with EHMTs [167]. Elevated levels of CDYL2 are associated with poor clinical outcomes in ER^+^ breast cancer. The EHMT-CDYL2 complex localizes on the *miR-124* gene promoter, suppressing its transcription by depositing H3K9me2 marks [167]. The overexpression of CDYL2 induces EMT and CSC maintenance in MCF7 and MDA-MB-231 breast cancer cells by increasing the Ser536 phosphorylation of p65 and STAT3 activity by Tyr705 phosphorylation, implicating both the NF-kB and STAT3 signaling pathways [128,168]. The knockdown of CDYL2 or the administration of the G9a inhibitor UNC0642 were also shown to reinstate miR-124 expression, suppressing the migratory potential and sphere formation capability of MDA-MB-231 cells.

### 6.5. lncRNA

lncRNAs can act as protein scaffolds, bringing proteins in proximity through the formation of ribonucleoprotein complexes [169]. Through RNA immunoprecipitation (RIP), the lncRNAs TERNA1 and NEAT1 were shown to act as protein scaffolds to G9a, Snail and DNMT, bringing them in close proximity for the formation of the G9a-Snail-DNMT1 complex [129,130]. This complex is directed to E-cadherin promoters in HCC and osteosarcoma. The depletion of TERNA1 or NEAT1 inhibits G9a, Snail, and DNMT1 formation, decreasing DNA and H3K9 methylation and rescuing E-cadherin expression [129,130].

lncRNAs also inhibit the normal function of proteins by acting as decoy DNA sequences to bind and sequester proteins away from their target sites [170]. In glioblastoma multiforme (GBM), the HOTAIRM1 lncRNA oncogene, which is transcribed from the antisense direction of the HomeoboxA1 (*HOXA1*) gene, binds and sequesters G9a away from the *HOXA1* gene promoter [131]. As the HOXA1 transcription factor belongs to the Hox family of proteins that are involved in multiple signaling pathways, the sequestration of G9a prevents the G9a-mediated silencing of HOXA1, resulting in increased cell proliferation, migration, and invasion and a reduction in apoptosis [131].

## 7. Targeting Regulators and EHMT Interactors in Cancer

### 7.1. Upstream Regulators

#### 7.1.1. Copy Number Gains and Gain-of-Function Mutation

Genomic editing is a promising therapeutic tool for correcting cancers driven by G9a copy number gains or gain-of-function mutations. Three major genome editing technologies are the transcription activator-like effector nucleases (TALENs), zinc-finger nucleases (ZFNs), and clustered regularly interspaced short palindromic repeat (CRISPR)-Cas-associated nucleases [53]. These technologies are able to induce double-strand breaks (DSBs) at target sites before deleting the target DNA sequence [171]. A donor DNA sequence can then be artificially synthesized to act as a template for gene correction or gene addition at the region where the DSBs were generated [54]. While gene editing approaches have made it into anti-cancer clinical trials, the focus is on generating chimeric antigen receptor (CAR) T cells to attack malignant cells [55,56]. No studies to date have attempted to correct the genetic aberration in tumors that are likely due to the limitations in editing, target specificity, efficacy, and delivery [172,173].

#### 7.1.2. EGFR Signaling

An EGFR inhibitor may be effective in targeting EGFR-STAT3-G9a-driven cancers. EGFR, a tyrosine kinase transmembrane receptor, is overexpressed in multiple cancers [174]. Ligand binding causes the dimerization of the receptor, allowing transphosphorylation to occur. This then serves as a docking site for various cytoplasmic substates, triggering various signaling cascades, such as RAS-RAF-MEK, PI3K-AKT-mTOR, and Src-STAT3 [175].

EGFR inhibitors are classified into two broad categories: irreversible or reversible. Irreversible inhibitors such as Afatinib, Neratinib, and Dacomitinib covalently bind to a cysteine residue on EGFR [60]. In contrast, reversible EGFR inhibitors such as Erlotinib, Lapatinib, and Gefitinib compete with ATP for the ATP binding pocket on EGFR without establishing any covalent interaction [59,60]. In a recent study, Erlotinib and the HDAC inhibitor SAHA displayed synergistic efficacy against mucoepidermoid carcinoma [176]. A similar combinatory approach with EHMT inhibitors can be considered for cancers with aberrant EGFR signaling and EHMT expression.

#### 7.1.3. NR4A1

As NR4A1 transcriptionally regulates G9a expression, targeting NR4A1 may likewise be effective in therapeutically treating malignancies driven by the NR4A1-G9a axis. Targeting NR4A1 was shown to be effective in reducing tumor growth in multiple types of cancer, highlighting the importance of NR4A1 in driving tumor progression [177].

NR4A1 activity can be antagonized by 1,1-bis(3’-indolyl)-1-(p-substituted phenyl)methane (C-DIMs) analogs. C-DIM analogs bind to the ligand-binding domain of NR4A1, inhibiting NR4A1 transactivation activity [62]. The treatment of CRC and pancreatic cancer cells with *p*-hydroxyphenyl C-DIM (DIM-C-pPhOH), a C-DIM analog, leads to a reduction in cancer cell growth. In addition, NR4A1 is a downstream target of androgen signaling [178]. The treatment of prostate cancer cells with Lestaurtinib perturbed androgen signaling, directly decreasing the expression of NR4A1 and its target genes [63]. Hence, inhibiting NR4A1 may be effective in inhibiting G9a.

#### 7.1.4. miRNAs

As miR-122 specifically targets G9a mRNA, promoting miR-122 expression provides a handle to downregulate G9a expression. The promoter of miR-122 is often subjected to DNA hypermethylation by DNA methyltransferase 1 (DNMT1) [71]. 5-azacytidine (5-Aza) and decitabine, inhibitors of DNMT1, can increase the expression of miR-122 [71,72]. Additionally, a number of lncRNA have been shown to suppress miR-122 expression. The small nucleolar RNA host gene 7 (SNHG7) lncRNA interacts with miR-122, resulting in its degradation [179]. The homeobox transcript antisense intergenic RNA (HOTAIR) lncRNA also promotes the DNA methylation of the miR-122 promoter to silence miR-122 [72]. Therefore, targeting lncRNAs upstream of miR-122 could rescue miR-122 expression, thereby reducing G9a expression. Ribonuclease-targeting chimeras (RIBOTACs), a group of small molecules that are designed to bind with ribonucleases and mediate specific RNA degradation, present a promising new approach to target miRNAs [65,180].

#### 7.1.5. Post-Translational Modifiers

EHMTs are subject to inhibition through hydroxylation by FIH and PHD under normoxic conditions [181]. As PHD and FIH hydroxylase activities are impaired under hypoxic conditions, it may be possible to reduce the reactive oxygen species (ROS) that are increased during hypoxia [68] to induce FIH and PHD hydroxylase activities to inhibit EHMTs. This can be achieved by using compounds with antioxidant properties such as ascorbic acid, carotenoids, and tocopherol [67].

No known agonist of speckled-type POZ protein to promote EHMT degradation is currently available. The advent of PROTAC technology may provide a novel approach to drive SPOP-mediated protein degradation. PROTACs are small bifunctional molecules that bind to a target protein and an E3-ubiquitin ligase simultaneously [73]. This promotes the ubiquitination and proteasomal degradation of the target protein.

### 7.2. Targeting EHMT Interactors in Cancer

EHMTs interact with a diverse group of proteins. Due to their methyltransferase-activity-independent roles, targeting their interacting partners or specifically inhibiting the formation of EHMT complexes may be critical for EHMT-complex-driven malignancies. These strategies can also overcome existing limitations of current EHMT inhibitors.

However, no known protein–protein interaction (PPI) modulators that specifically target EHMT complexes or EHMT-binding domain(s) are currently available. PPI modulators can be small molecules, peptides, or antibodies [182]. Since EHMTs have been shown to complex with various factors to drive tumor formation, focusing on key interacting sites of EHMTs for the development of PPI modulators may be a worthwhile strategy to target EHMT-complex-driven malignancies.

In the meantime, an alternative is to target the interacting partners of EHMTs to potentially inhibit complex formation. In the following sections, we discuss the possibilities of targeting EHMT interactors specifically through available inhibitors and evaluate their potential in disrupting EHMT complexes.

### 7.3. Targeting Transcription Factors

#### 7.3.1. Inhibition of MDM2/Activation of p53

There are currently no known inhibitors specifically targeting the EHMT-p53 interaction. However, as EHMTs inactivate p53, which is further augmented by MDM2, targeting MDM2 may dampen EHMT-mediated p53 degradation. Nutlin analogs are a pioneer class of small molecule inhibitors that inhibit the MDM2-p53 interaction [87]. MI-219 is a recently generated small molecule MDM2 inhibitor generated from a class of chemicals known as spirooxindole. MI-219 showed excellent binding to MDM2 and can disrupt the MDM2-p53 interaction [88]. Additionally, MI-219 has favorable pharmacological properties, including 55% oral bioavailability observed in mice and great affinity towards MDM2 [183]. Nonetheless, mapping the key domains for the MDM2-EHMTs-p53 complex is still needed for the discovery of novel inhibitors that specifically target the MDM2-EHMTs-p53 complex.

#### 7.3.2. Inhibition of Myc

As G9a interacts with Myc to promote proliferation in cancer cells by binding to the promoter regions of target genes, inhibiting Myc may impede the repressive function of this complex. Small molecule inhibitors of Myc either disrupt Myc/Max dimerization or block the binding to the E-box element on gene promoters [184,185]. Despite being one of the most well-studied oncogenes, no small molecule inhibitor against Myc has made it to clinical trials due to poor target selectivity, non-specificity in differentiating cancer cells from normal cells, and low potency [92].

Among all reported Myc inhibitors, KJ-pyr-9 was found to have the highest binding affinity for Myc and was most effective in inhibiting human triple-negative breast cancer both in vitro and in vivo [186]. KJ-pyr-9 inhibits Myc by blocking its ability to dimerize with Max. JKY-2-169, a synthetic α-helix mimetic small molecule inhibitor, can also inhibit Myc by disrupting the Myc/Max complex [91]. The disruption of the complex prevents the Myc/Max heterodimer from binding to the E-box element. Besides small molecules inhibitors, synthetic peptides and proteins are alternative inhibitors. Omomyc, a 91-residue mini mutant c-Myc dominant negative protein, dimerizes with wild-type c-Myc and Max, impeding wild-type Myc dimerization and disrupting the Myc regulatory network [187]. Preliminary studies have shown that Omomyc is effective in multiple cancer models such as breast, lung, brain, and pancreatic cancers [92].

However, small molecule inhibitors and Omomyc target the C-terminal basic helix-loop-helix leucine zipper domain (bHLHLZ) instead of the MYC box II domain, which is important for the G9a-MYC interaction. Nonetheless, inhibiting Myc and, by extension, Myc/Max dimerization could potentially inhibit the activity of the G9a-Myc complex, as it prevents Myc from directing G9a towards Myc-repressed target genes and therefore blocks the ability of G9a to repress common target genes.

Instead of targeting Myc, another approach is to decrease Myc expression by targeting its upstream regulators, thereby disrupting G9a-Myc complex formation due to the lack of Myc protein. For example, using a combination treatment of JQ1, an inhibitor for BRD4, and NSC, an inhibitor of RAC1, leads to a decrease in Myc expression and, in turn, reduces G9a-Myc complex formation in HER-2 and triple-negative breast cancer [188].

#### 7.3.3. Inhibition of STAT3

As activated STAT3 interacts with G9a, forming a repressive complex, inhibiting STAT3 may prevent the activity of this complex. Current small molecule inhibitors generally inhibit STAT3 activity by binding to the SH2 domain, preventing STAT3 from phosphorylation or blocking STAT3 DNA binding [189].

STA-21, a quinone analogue, is a small molecule inhibitor that binds to the SH2 domain of STAT3, preventing STAT3 dimerization and DNA binding activity while having little effect on STAT3 phosphorylation [98]. Stattic is another small molecule inhibitor that binds to the SH2 domain of STAT3. Stattic forms hydrogen bonds with Arg 609, Ser 611, and Ser 613 to create a barrier that prevents Tyr 705 from phosphorylation, keeping STAT3 in an inactive state [99].

Platinum compounds have been shown to impede STAT3′s DNA binding activity. For instance, the chemotherapeutic drug cisplatin and the platinum compound IS3295 can selectively inhibit STAT3 by attaching to STAT3 and interrupting the binding of activated STAT3 to DNA [100]. SH003, a STAT3 inhibitor, disrupts the G9a-STAT3 interaction by preventing STAT3 phosphorylation and nuclear localization [97,190]. This suggests that blocking STAT3 phosphorylation and nuclear localization could potentially inhibit the formation and activity of the G9a-STAT3 complex.

#### 7.3.4. Activation of FOXO1

Since SKP2 degrades FOXO1 upon methylation by G9a, targeting SKP2 activity may enhance FOXO1 activity despite increased G9a activity in cancers. The expression of SKP2 can be disrupted by preventing the SKP2-SCF complex [191]. Cpda, a compound identified from a high-throughput screen, prevented the interaction between Skp2 and SCF and blocked the proliferation of neoplastic cells [192]. As PPARγ is an upstream negative regulator of SKP2, increasing the activity of PPARγ can reduce SKP2 expression [193]. Troglitazone, a PPARγ agonist, has been used to treat SKP2-overexpressing HCC [102]. A decrease in SKP2 expression along with cell cycle arrest were observed in HCC cells upon treatment with troglitazone. Natural compounds such as gallic acid, EGCG, and tea leaf extracts have also been found to inhibit SKP2 expression [103]. The compound SZL-P1-41, which has been identified to directly interact with the F-box domain of SKP2, can block the SKP1 interaction for the formation of the SKP2-SCF complex [194].

Another way of inhibiting SKP2 activity is by blocking the SKP2-CKS1 interaction to prevent the transfer of ubiquitin to target proteins. High-throughput screens revealed several compounds such as 22d, Skp2E3LIs, NSC689857, and LinichlorinA that are effective in interfering with the SKP2-CKS1 interaction [104,105,106,195]. As a result, the targeting of SKP2 may diminish G9a-dependent FOXO1 degradation by decreasing the ubiquitination of FOXO1.

### 7.4. Targeting Zinc Finger Transcription Factors

G9a interacts with Snail and DNMT1 to form a complex responsible for inducing EMT in cancers; hence, targeting Snail may prevent complex formation to inhibit the EMT transition. An oligonucleotide-conjugated Co(III) complex was derived to selectively inhibit Snail family transcription factors by preventing DNA binding while maintaining the ability of other transcription factors to bind to DNA [196]. Inhibiting Snail DNA-binding capability prevents the complex from binding to downstream targets.

Additionally, as expression of Snail and Slug are transcriptionally regulated by TGF-β signaling, targeting TGF-β can reduce Snail and Slug expression [197,198]. Current available inhibitors of TGF-β signaling include small molecules that target TGF-β receptor kinases, monoclonal antibodies that impede ligand–receptor binding, and antisense oligonucleotides that block the translation of key players of the TGF-β signaling pathway [199]. A majority of the TGF-β inhibitors are designed as ATP mimetics to compete with ATP for the ATP binding site of TGFβRI kinase and inhibit the catalytic activity of TGFβ1R [199]. These small molecule inhibitors include SD-093 and LY2157299, which block TGF-β-induced cancer cell migration and EMT [112,113]. Monoclonal antibodies targeting the TGF-β pathway aim to disrupt the interaction between the TGF-β ligand and the TGF-β receptor, thereby effectively impeding ligand signaling [113]. For example, GC-1008, a pan monoclonal antibody, is able to neutralize all three TGF-β ligand isoforms and has been tested in clinical trials against renal cell carcinoma [200]. AP12009 and ISTH0036 are the two reported antisense oligonucleotides that target the TGF-β2 ligand [114,116].

Since Snail and Slug are downstream targets, the inhibition of TGF-β signaling can result in a decrease in the expression of both Snail and Slug. Because they act as chaperones, decreasing their expression may disrupt complex formation with G9a, thus relieving the repressive effect on target genes such as E-cadherin.

### 7.5. Targeting Non-Transcription Factors

#### Inhibition of Cyclin D

As cyclin D1 acts as a chaperone for G9a to target genes, therapeutic agents against cyclin D that induce its degradation or prevent its transcription may be beneficial in targeting the function of the cyclin D-G9a complex. The histone deacetylase inhibitor, trichostatin A (TSA), can promote the ubiquitin-dependent proteasomal degradation of cyclin D1 by upregulating the Skp2-SCF E3 ligase complex [123]. Retinoic acid (Vitamin A) has been shown to induce cyclin D1 proteolysis via a ubiquitin-dependent proteasome degradation pathway [201]. S14161 decreases the mRNA expression of cyclin D1, *2,* and 3 by suppressing the activity of PI3K [202]. Glucocorticoids were also shown to reduce cyclin D1 expression by promoting the expression of SP1 transcription factor, which in turn induced the degradation of the MAF BZIP transcription factor (c-maf) [203]. The c-maf oncogene is known to induce cyclin D transcription [204]. A natural product, kinetin riboside, represses the transcription of cyclin D1 and 2 by increasing the expression of the transcription repressor CREM [205]. Although it remains to be proven, it is likely that the decrease in cyclin D1 can reduce the availability of free cyclin D1 to bind to G9a, dampening G9a-cyclin D1 complex formation.

### 7.6. Targeting Epigenetic Regulators

#### Inhibition of CDYL

CDYL also functions as a chaperone that guides EHMTs to target regions on the chromatin. Yang and colleagues have identified the first selective small-molecule inhibitor of CDYL, benzo [d]oxazol-2(3H)-one (compound D03). Mechanistically, compound D03 engages with CDYL and hinders CDYL binding to the target region on the chromatin, thereby preventing CDYL from repressing target genes [127]. Compound D03 could be effective in antagonizing the CDYL-G9a complex. Since G9a does not have a DNA binding domain, compound D03 may effectively block the G9a-CDYL complex from binding to target gene promoters, thus eliminating G9a’s repressive role on target genes.

## 8. Conclusions

EHMTs are important epigenetic regulators that are required for normal physiological processes. The dysregulation of EHMTs and their complexes are common features associated with multiple cancers, highlighting the need to therapeutically target these proteins. Although substantial work has been conducted on the downstream effectors of EHMTs, the upstream mechanisms responsible for the altered expression of EHMTs and their interacting partners are not well elucidated. Because current inhibitors are limited by their various physiochemical properties or selectivity profiles, alternative approaches are required to therapeutically target EHMTs and their complexes. In this review, we present an overview of the upstream regulators of EHMTs and offer insights into the possibilities of therapeutically targeting its regulators to dampen EHMT expression. We also present an overview of EHMTs’ interacting partners and evaluate the potential of available inhibitors against these interactors to suppress EHMT complex formation. Nonetheless, these approaches are not without their caveats. Because EHMTs lie upstream of many gene networks, targeting the expression of EHMTs or their interacting partners could indirectly antagonize the beneficial role of the regulator or pathways, resulting in many unwanted side effects. This is especially true for pathways or proteins of pleiotropic nature such as Snail/Slug and STAT3, as their functions depend on the genetic background and biochemical context of the cells. The use of a combinatorial treatment through the careful optimization of EHMT inhibitors and chemotherapeutic agents may possibly overcome this challenge.

Instead of specifically targeting individual complexes in various cancers, the development of PPI modulators against the SET domain may be another viable approach, as most of the interactions involve this domain. Still, there is a need to consider the specificity of the PPI modulator, as it may block interactions with other proteins that are not involved in the oncogenesis. Therefore, targeting epigenetic regulators in cancer therapy requires a detailed evaluation of their networks. The PROTAC technology has great potential in driving EHMT proteasomal degradation in cancers. As the understanding of the epigenetic landscape widens, the next generation of drugs that leverage the unique features of epigenetic regulators can be developed.

## Figures and Tables

**Table 1:** EHMT1/2 regulators in distinct cancers and potential therapeutic strategies are summarized.

Upstream Regulators	Molecule	Disease	Phenotype	Potential Therapeutics
**Genetic dysregulation**				
Copy number gains	G9a (6p21)	Melanoma [52]	Proliferation	Gene therapy (yet to be explored) [53,54]
		HCC [27]	Proliferation and migration	
Gain of function	G9a (Glycine 1069)	Melanoma [52]	Proliferation	Mutant-specific inhibition (yet to be explored) [55,56]
		CRC [57]	Migration and invasion	
**Transcriptional Dysregulation**				
EGFR	G9a	Breast cancer [58]	Proliferation and survival	Lapatinib [59] Neratinib [60]
STAT3	G9a	Breast cancer [58]	Proliferation and survival	BB1608 [58]
NR4A1	G9a	ARMS [61] Breast cancer [61] Lung cancer [61]	Proliferation, Tumorigenesis	CDIM8 [62] Diindolylmethane analogues [62] Lestaurtinib [63]
miR-122	G9a	HCC [27,64]	Reduces invasion and survival	RIBOTACS [65]
miR-1				
**Post-translational dysregulation**				
FIH	G9a/GLP	Ovarian cancer [66]	Reduce migration and dissemination	Carotenoids [67] Ascorbic acid [68]
PHD1	G9a	Breast cancer [69]	Reduce proliferation and metastasis	Tocopherol [67]
SPOP	GLP	Prostate cancer [70]	Reduce proliferation and survival	Potential activation by DNMT inhibitors [71,72] PROTAC [73]
APC/C^cdh1^	G9a/GLP	Cancers [74]	senescence	PROTAC [73]
ATM	G9a	Cancers [75]	DNA repair	-

## Data Availability

Not applicable.

## References

[1] Maleszewska M., Wojtas B., Kaminska B. (2018). Deregulation of Epigenetic Mechanisms in Cancer. Postępy Biochem..

[2] Zhang Z., Zhang R. (2015). Epigenetics in Autoimmune Diseases: Pathogenesis and Prospects for Therapy. Autoimmun. Rev..

[3] Mirabella A.C., Foster B.M., Bartke T. (2016). Chromatin Deregulation in Disease. Chromosoma.

[4] Zhao Z., Shilatifard A. (2019). Epigenetic Modifications of Histones in Cancer. Genome Biol..

[5] Murray K. (1964). The Occurrence of Iε-N-Methyl Lysine in Histones. Biochemistry.

[6] Bannister A.J., Kouzarides T. (2011). Regulation of Chromatin by Histone Modifications. Cell Res..

[7] Husmann D., Gozani O. (2019). Histone Lysine Methyltransferases in Biology and Disease. Nat. Struct. Mol. Biol..

[8] Tachibana M., Sugimoto K., Fukushima T., Shinkai Y. (2001). SET Domain-Containing Protein, G9a, Is a Novel Lysine-Preferring Mammalian Histone Methyltransferase with Hyperactivity and Specific Selectivity to Lysines 9 and 27 of Histone H3. J. Biol. Chem..

[9] Rice J.C., Briggs S.D., Ueberheide B., Barber C.M., Shabanowitz J., Hunt D.F., Shinkai Y., Allis C.D. (2003). Histone Methyltransferases Direct Different Degrees of Methylation to Define Distinct Chromatin Domains. Mol. Cell.

[10] Bittencourt D., Wu D.-Y., Jeong K.W., Gerke D.S., Herviou L., Ianculescu I., Chodankar R., Siegmund K.D., Stallcup M.R. (2012). G9a Functions as a Molecular Scaffold for Assembly of Transcriptional Coactivators on a Subset of Glucocorticoid Receptor Target Genes. Proc. Natl. Acad. Sci. USA.

[11] Michalak E.M., Visvader J.E. (2016). Dysregulation of Histone Methyltransferases in Breast Cancer—Opportunities for New Targeted Therapies?. Mol. Oncol..

[12] Pangeni R.P., Yang L., Zhang K., Wang J., Li W., Guo C., Yun X., Sun T., Wang J., Raz D.J. (2020). G9a Regulates Tumorigenicity and Stemness through Genome-Wide DNA Methylation Reprogramming in Non-Small Cell Lung Cancer. Clin. Epigenetics.

[13] Souza B.K., Freire N.H., Jaeger M., de Farias C.B., Brunetto A.L., Brunetto A.T., Roesler R. (2021). EHMT2/G9a as an Epigenetic Target in Pediatric and Adult Brain Tumors. Int. J. Mol. Sci..

[14] Hua K.-T., Wang M.-Y., Chen M.-W., Wei L.-H., Chen C.-K., Ko C.-H., Jeng Y.-M., Sung P.-L., Jan Y.-H., Hsiao M. (2014). The H3K9 Methyltransferase G9a Is a Marker of Aggressive Ovarian Cancer That Promotes Peritoneal Metastasis. Mol. Cancer.

[15] Casciello F., Windloch K., Gannon F., Lee J.S. (2015). Functional Role of G9a Histone Methyltransferase in Cancer. Front. Immunol..

[16] Kondo Y., Shen L., Ahmed S., Boumber Y., Sekido Y., Haddad B.R., Issa J.-P.J. (2008). Downregulation of Histone H3 Lysine 9 Methyltransferase G9a Induces Centrosome Disruption and Chromosome Instability in Cancer Cells. PLoS ONE.

[17] Rada M., Vasileva E., Lezina L., Marouco D., Antonov A.V., Macip S., Melino G., Barlev N.A. (2017). Human EHMT2/G9a Activates P53 through Methylation-Independent Mechanism. Oncogene.

[18] Cao H., Li L., Yang D., Zeng L., Yewei X., Yu B., Liao G., Chen J. (2019). Recent Progress in Histone Methyltransferase (G9a) Inhibitors as Anticancer Agents. Eur. J. Med. Chem..

[19] Jan S., Dar M.I., Wani R., Sandey J., Mushtaq I., Lateef S., Syed S.H. (2021). Targeting EHMT2/G9a for Cancer Therapy: Progress and Perspective. Eur. J. Pharmacol..

[20] Tachibana M., Ueda J., Fukuda M., Takeda N., Ohta T., Iwanari H., Sakihama T., Kodama T., Hamakubo T., Shinkai Y. (2005). Histone Methyltransferases G9a and GLP Form Heteromeric Complexes and Are Both Crucial for Methylation of Euchromatin at H3-K9. Genes Dev..

[21] Poulard C., Noureddine L.M., Pruvost L., Le Romancer M. (2021). Structure, Activity, and Function of the Protein Lysine Methyltransferase G9a. Life.

[22] Trievel R.C., Beach B.M., Dirk L.M.A., Houtz R.L., Hurley J.H. (2002). Structure and Catalytic Mechanism of a SET Domain Protein Methyltransferase. Cell.

[23] Collins R.E., Northrop J.P., Horton J.R., Lee D.Y., Zhang X., Stallcup M.R., Cheng X. (2008). The Ankyrin Repeats of G9a and GLP Histone Methyltransferases Are Mono- and Dimethyllysine Binding Modules. Nat. Struct. Mol. Biol..

[24] Estève P.-O., Patnaik D., Chin H.G., Benner J., Teitell M.A., Pradhan S. (2005). Functional Analysis of the N- and C-Terminus of Mammalian G9a Histone H3 Methyltransferase. Nucleic Acids Res..

[25] Shinkai Y., Tachibana M. (2011). H3K9 Methyltransferase G9a and the Related Molecule GLP. Genes Dev..

[26] Ling B.M.T., Bharathy N., Chung T.-K., Kok W.K., Li S., Tan Y.H., Rao V.K., Gopinadhan S., Sartorelli V., Walsh M.J. (2012). Lysine Methyltransferase G9a Methylates the Transcription Factor MyoD and Regulates Skeletal Muscle Differentiation. Proc. Natl. Acad. Sci. USA.

[27] Wei L., Chiu D.K.-C., Tsang F.H.-C., Law C.-T., Cheng C.L.-H., Au S.L.-K., Lee J.M.-F., Wong C.C.-L., Ng I.O.-L., Wong C.-M. (2017). Histone Methyltransferase G9a Promotes Liver Cancer Development by Epigenetic Silencing of Tumor Suppressor Gene RARRES3. J. Hepatol..

[28] Dang N.-N., Jiao J., Meng X., An Y., Han C., Huang S. (2020). Abnormal Overexpression of G9a in Melanoma Cells Promotes Cancer Progression via Upregulation of the Notch1 Signaling Pathway. Aging.

[29] Hu L., Zang M., Wang H., Zhang B., Wang Z., Fan Z., Wu H., Li J., Su L., Yan M. (2018). G9A Promotes Gastric Cancer Metastasis by Upregulating ITGB3 in a SET Domain-Independent Manner. Cell Death Dis..

[30] Jin Y., Park S., Park S.-Y., Lee C.-Y., Eum D.-Y., Shim J.-W., Choi S.-H., Choi Y.-J., Park S.-J., Heo K. (2022). G9a Knockdown Suppresses Cancer Aggressiveness by Facilitating Smad Protein Phosphorylation through Increasing BMP5 Expression in Luminal A Type Breast Cancer. Int. J. Mol. Sci..

[31] Liu S., Ye D., Guo W., Yu W., He Y., Hu J., Wang Y., Zhang L., Liao Y., Song H. (2015). G9a Is Essential for EMT-Mediated Metastasis and Maintenance of Cancer Stem Cell-like Characters in Head and Neck Squamous Cell Carcinoma. Oncotarget.

[32] Hsiao S.-M., Chen M.-W., Chen C.-A., Chien M.-H., Hua K.-T., Hsiao M., Kuo M.-L., Wei L.-H. (2015). The H3K9 Methyltransferase G9a Represses E-Cadherin and Is Associated with Myometrial Invasion in Endometrial Cancer. Ann. Surg. Oncol..

[33] Sun T., Zhang K., Pangeni R.P., Wu J., Li W., Du Y., Guo Y., Chaurasiya S., Arvanitis L., Raz D.J. (2021). G9a Promotes Invasion and Metastasis of Non-Small Cell Lung Cancer through Enhancing Focal Adhesion Kinase Activation via NF-ΚB Signaling Pathway. Mol. Cancer Res. MCR.

[34] Rowbotham S.P., Li F., Dost A.F.M., Louie S.M., Marsh B.P., Pessina P., Anbarasu C.R., Brainson C.F., Tuminello S.J., Lieberman A. (2018). H3K9 Methyltransferases and Demethylases Control Lung Tumor-Propagating Cells and Lung Cancer Progression. Nat. Commun..

[35] Guan X., Zhong X., Men W., Gong S., Zhang L., Han Y. (2014). Analysis of EHMT1 Expression and Its Correlations with Clinical Significance in Esophageal Squamous Cell Cancer. Mol. Clin. Oncol..

[36] Cheng C.-C., Chang J., Huang S.C.-C., Lin H.-C., Ho A.-S., Lim K.-H., Chang C.-C., Huang L., Chang Y.-C., Chang Y.-F. (2017). YM155 as an Inhibitor of Cancer Stemness Simultaneously Inhibits Autophosphorylation of Epidermal Growth Factor Receptor and G9a-Mediated Stemness in Lung Cancer Cells. PLoS ONE.

[37] Yang Y., Shen J., Yan D., Yuan B., Zhang S., Wei J., Du T. (2018). Euchromatic Histone Lysine Methyltransferase 1 Regulates Cancer Development in Human Gastric Cancer by Regulating E-Cadherin. Oncol. Lett..

[38] Nachiyappan A., Soon J.L.J., Lim H.J., Lee V.K., Taneja R. (2022). EHMT1 Promotes Tumor Progression and Maintains Stemness by Regulating ALDH1A1 Expression in Alveolar Rhabdomyosarcoma. J. Pathol..

[39] Nachiyappan A., Gupta N., Taneja R. (2022). EHMT1/EHMT2 in EMT, Cancer Stemness and Drug Resistance: Emerging Evidence and Mechanisms. FEBS J..

[40] Wen B., Wu H., Shinkai Y., Irizarry R.A., Feinberg A.P. (2009). Large Histone H3 Lysine 9 Dimethylated Chromatin Blocks Distinguish Differentiated from Embryonic Stem Cells. Nat. Genet..

[41] Berman B.P., Weisenberger D.J., Aman J.F., Hinoue T., Ramjan Z., Liu Y., Noushmehr H., Lange C.P.E., Van Dijk C.M., Tollenaar R.A.E.M. (2012). Regions of Focal DNA Hypermethylation and Long-Range Hypomethylation in Colorectal Cancer Coincide with Nuclear Lamina–Associated Domains. Nat. Genet..

[42] Hansen K.D., Timp W., Bravo H.C., Sabunciyan S., Langmead B., McDonald O.G., Wen B., Wu H., Liu Y., Diep D. (2011). Increased Methylation Variation in Epigenetic Domains across Cancer Types. Nat. Genet..

[43] McDonald O.G., Wu H., Timp W., Doi A., Feinberg A.P. (2011). Genome-Scale Epigenetic Reprogramming during Epithelial-to-Mesenchymal Transition. Nat. Struct. Mol. Biol..

[44] Rugo H.S., Jacobs I., Sharma S., Scappaticci F., Paul T.A., Jensen-Pergakes K., Malouf G.G. (2020). The Promise for Histone Methyltransferase Inhibitors for Epigenetic Therapy in Clinical Oncology: A Narrative Review. Adv. Ther..

[45] Yuan Y., Wang Q., Paulk J., Kubicek S., Kemp M.M., Adams D.J., Shamji A.F., Wagner B.K., Schreiber S.L. (2012). A Small-Molecule Probe of the Histone Methyltransferase G9a Induces Cellular Senescence in Pancreatic Adenocarcinoma. ACS Chem. Biol..

[46] Halby L., Champion C., Sénamaud-Beaufort C., Ajjan S., Drujon T., Rajavelu A., Ceccaldi A., Jurkowska R., Lequin O., Nelson W.G. (2012). Rapid Synthesis of New DNMT Inhibitors Derivatives of Procainamide. Chembiochem Eur. J. Chem. Biol..

[47] Kubicek S., O’Sullivan R.J., August E.M., Hickey E.R., Zhang Q., Teodoro M.L., Rea S., Mechtler K., Kowalski J.A., Homon C.A. (2007). Reversal of H3K9me2 by a Small-Molecule Inhibitor for the G9a Histone Methyltransferase. Mol. Cell.

[48] Liu F., Chen X., Allali-Hassani A., Quinn A.M., Wasney G.A., Dong A., Barsyte D., Kozieradzki I., Senisterra G., Chau I. (2009). Discovery of a 2,4-Diamino-7-Aminoalkoxyquinazoline as a Potent and Selective Inhibitor of Histone Lysine Methyltransferase G9a. J. Med. Chem..

[49] Liu F., Chen X., Allali-Hassani A., Quinn A.M., Wigle T.J., Wasney G.A., Dong A., Senisterra G., Chau I., Siarheyeva A. (2010). Protein Lysine Methyltransferase G9a Inhibitors: Design, Synthesis, and Structure Activity Relationships of 2,4-Diamino-7-Aminoalkoxy-Quinazolines. J. Med. Chem..

[50] Vedadi M., Barsyte-Lovejoy D., Liu F., Rival-Gervier S., Allali-Hassani A., Labrie V., Wigle T.J., Dimaggio P.A., Wasney G.A., Siarheyeva A. (2011). A Chemical Probe Selectively Inhibits G9a and GLP Methyltransferase Activity in Cells. Nat. Chem. Biol..

[51] Deimling S.J., Olsen J.B., Tropepe V. (2017). The Expanding Role of the Ehmt2/G9a Complex in Neurodevelopment. Neurogenesis.

[52] Kato S., Weng Q.Y., Insco M.L., Chen K.Y., Muralidhar S., Pozniak J., Diaz J.M.S., Drier Y., Nguyen N., Lo J.A. (2020). Gain-of-Function Genetic Alterations of G9a Drive Oncogenesis. Cancer Discov..

[53] Gaj T., Sirk S.J., Shui S., Liu J. (2016). Genome-Editing Technologies: Principles and Applications. Cold Spring Harb. Perspect. Biol..

[54] Kim H., Kim J.-S. (2014). A Guide to Genome Engineering with Programmable Nucleases. Nat. Rev. Genet..

[55] Li H., Yang Y., Hong W., Huang M., Wu M., Zhao X. (2020). Applications of Genome Editing Technology in the Targeted Therapy of Human Diseases: Mechanisms, Advances and Prospects. Signal Transduct. Target. Ther..

[56] Cyranoski D. (2016). CRISPR Gene-Editing Tested in a Person for the First Time. Nature.

[57] Bergin C.J., Benoit Y.D. (2020). G9a Is SETting the Stage for Colorectal Oncogenesis. Genes.

[58] Chang Y.-F., Lim K.-H., Chiang Y.-W., Sie Z.-L., Chang J., Ho A.-S., Cheng C.-C. (2019). STAT3 Induces G9a to Exacerbate HER3 Expression for the Survival of Epidermal Growth Factor Receptor-Tyrosine Kinase Inhibitors in Lung Cancers. BMC Cancer.

[59] Ayati A., Moghimi S., Salarinejad S., Safavi M., Pouramiri B., Foroumadi A. (2020). A Review on Progression of Epidermal Growth Factor Receptor (EGFR) Inhibitors as an Efficient Approach in Cancer Targeted Therapy. Bioorganic Chem..

[60] Abourehab M.A.S., Alqahtani A.M., Youssif B.G.M., Gouda A.M. (2021). Globally Approved EGFR Inhibitors: Insights into Their Syntheses, Target Kinases, Biological Activities, Receptor Interactions, and Metabolism. Molecules.

[61] Shrestha R., Mohankumar K., Jin U.-H., Martin G., Safe S. (2021). The Histone Methyltransferase Gene G9A Is Regulated by Nuclear Receptor 4A1 in Alveolar Rhabdomyosarcoma Cells. Mol. Cancer Ther..

[62] Lee S.-O., Li X., Hedrick E., Jin U.-H., Tjalkens R.B., Backos D.S., Li L., Zhang Y., Wu Q., Safe S. (2014). Diindolylmethane Analogs Bind NR4A1 and Are NR4A1 Antagonists in Colon Cancer Cells. Mol. Endocrinol..

[63] Köhler J., Erlenkamp G., Eberlin A., Rumpf T., Slynko I., Metzger E., Schüle R., Sippl W., Jung M. (2012). Lestaurtinib Inhibits Histone Phosphorylation and Androgen-Dependent Gene Expression in Prostate Cancer Cells. PLoS ONE.

[64] Yuan L.-T., Lee W.-J., Yang Y.-C., Chen B.-R., Yang C.-Y., Chen M.-W., Chen J.-Q., Hsiao M., Chien M.-H., Hua K.-T. (2021). Histone Methyltransferase G9a-Promoted Progression of Hepatocellular Carcinoma Is Targeted by Liver-Specific Hsa-MiR-122. Cancers.

[65] Kargbo R.B. (2021). RIBOTACs: Small Molecules Selectively Destroy Cancer-Associated RNA. ACS Med. Chem. Lett..

[66] Kang J., Shin S.-H., Yoon H., Huh J., Shin H.-W., Chun Y.-S., Park J.-W. (2018). FIH Is an Oxygen Sensor in Ovarian Cancer for G9a/GLP-Driven Epigenetic Regulation of Metastasis-Related Genes. Cancer Res..

[67] Fuchs-Tarlovsky V. (2013). Role of Antioxidants in Cancer Therapy. Nutrition.

[68] Hielscher A., Gerecht S. (2015). Hypoxia and Free Radicals: Role in Tumor Progression and the Use of Engineering-Based Platforms to Address These Relationships. Free Radic. Biol. Med..

[69] Casciello F., Al-Ejeh F., Kelly G., Brennan D.J., Ngiow S.F., Young A., Stoll T., Windloch K., Hill M.M., Smyth M.J. (2017). G9a Drives Hypoxia-Mediated Gene Repression for Breast Cancer Cell Survival and Tumorigenesis. Proc. Natl. Acad. Sci. USA.

[70] Zhang J., Gao K., Xie H., Wang D., Zhang P., Wei T., Yan Y., Pan Y., Ye W., Chen H. (2021). SPOP Mutation Induces DNA Methylation via Stabilizing GLP/G9a. Nat. Commun..

[71] Coulouarn C., Factor V.M., Andersen J.B., Durkin M.E., Thorgeirsson S.S. (2009). Loss of MiR-122 Expression in Liver Cancer Correlates with Suppression of the Hepatic Phenotype and Gain of Metastatic Properties. Oncogene.

[72] Cheng D., Deng J., Zhang B., He X., Meng Z., Li G., Ye H., Zheng S., Wei L., Deng X. (2018). LncRNA HOTAIR Epigenetically Suppresses MiR-122 Expression in Hepatocellular Carcinoma via DNA Methylation. EBioMedicine.

[73] Neklesa T.K., Winkler J.D., Crews C.M. (2017). Targeted Protein Degradation by PROTACs. Pharmacol. Ther..

[74] Coppé J.-P., Desprez P.-Y., Krtolica A., Campisi J. (2010). The Senescence-Associated Secretory Phenotype: The Dark Side of Tumor Suppression. Annu. Rev. Pathol..

[75] Ginjala V., Rodriguez-Colon L., Ganguly B., Gangidi P., Gallina P., Al-Hraishawi H., Kulkarni A., Tang J., Gheeya J., Simhadri S. (2017). Protein-Lysine Methyltransferases G9a and GLP1 Promote Responses to DNA Damage. Sci. Rep..

[76] Liu L., Kimball S., Liu H., Holowatyj A., Yang Z.-Q. (2014). Genetic Alterations of Histone Lysine Methyltransferases and Their Significance in Breast Cancer. Oncotarget.

[77] Ma Y., Zhang H.-Y., Fei L.-R., Zhang M.-Y., Wang C.-C., Luo Y., Han Y.-C. (2018). SATB2 Suppresses Non-Small Cell Lung Cancer Invasiveness by G9a. Clin. Exp. Med..

[78] Chin H.G., Estève P.-O., Pradhan M., Benner J., Patnaik D., Carey M.F., Pradhan S. (2007). Automethylation of G9a and Its Implication in Wider Substrate Specificity and HP1 Binding. Nucleic Acids Res..

[79] Poulard C., Baulu E., Lee B.H., Pufall M.A., Stallcup M.R. (2018). Increasing G9a Automethylation Sensitizes B Acute Lymphoblastic Leukemia Cells to Glucocorticoid-Induced Death. Cell Death Dis..

[80] Wyld L., Bellantuono I., Tchkonia T., Morgan J., Turner O., Foss F., George J., Danson S., Kirkland J.L. (2020). Senescence and Cancer: A Review of Clinical Implications of Senescence and Senotherapies. Cancers.

[81] DNA Damage Signaling Triggers Degradation of Histone Methyltransferases through APC/C(Cdh1) in Senescent Cells—PubMed. https://pubmed.ncbi.nlm.nih.gov/22178396/.

[82] Srinivasan S., Shankar S.R., Wang Y., Taneja R. (2019). SUMOylation of G9a Regulates Its Function as an Activator of Myoblast Proliferation. Cell Death Dis..

[83] Scheer S., Zaph C. (2017). The Lysine Methyltransferase G9a in Immune Cell Differentiation and Function. Front. Immunol..

[84] Purcell D.J., Jeong K.W., Bittencourt D., Gerke D.S., Stallcup M.R. (2011). A Distinct Mechanism for Coactivator versus Corepressor Function by Histone Methyltransferase G9a in Transcriptional Regulation. J. Biol. Chem..

[85] Oh S.-T., Kim K.-B., Chae Y.-C., Kang J.-Y., Hahn Y., Seo S.-B. (2014). H3K9 Histone Methyltransferase G9a-Mediated Transcriptional Activation of P21. FEBS Lett..

[86] Chen L., Li Z., Zwolinska A.K., Smith M.A., Cross B., Koomen J., Yuan Z.-M., Jenuwein T., Marine J.-C., Wright K.L. (2010). MDM2 Recruitment of Lysine Methyltransferases Regulates P53 Transcriptional Output. EMBO J..

[87] Beloglazkina A., Zyk N., Majouga A., Beloglazkina E. (2020). Recent Small-Molecule Inhibitors of the P53–MDM2 Protein–Protein Interaction. Molecules.

[88] Shangary S., Wang S. (2009). Small-Molecule Inhibitors of the MDM2-P53 Protein-Protein Interaction to Reactivate P53 Function: A Novel Approach for Cancer Therapy. Annu. Rev. Pharmacol. Toxicol..

[89] Zhang J., Wang Y., Shen Y., He P., Ding J., Chen Y. (2018). G9a Stimulates CRC Growth by Inducing P53 Lys373 Dimethylation-Dependent Activation of *Plk1*. Theranostics.

[90] Schlereth K., Beinoraviciute-Kellner R., Zeitlinger M.K., Bretz A.C., Sauer M., Charles J.P., Vogiatzi F., Leich E., Samans B., Eilers M. (2010). DNA Binding Cooperativity of P53 Modulates the Decision between Cell-Cycle Arrest and Apoptosis. Mol. Cell.

[91] Jung K.-Y., Wang H., Teriete P., Yap J.L., Chen L., Lanning M.E., Hu A., Lambert L.J., Holien T., Sundan A. (2015). Perturbation of the C-Myc–Max Protein–Protein Interaction via Synthetic α-Helix Mimetics. J. Med. Chem..

[92] Madden S.K., De Araujo A.D., Gerhardt M., Fairlie D.P., Mason J.M. (2021). Taking the Myc out of Cancer: Toward Therapeutic Strategies to Directly Inhibit c-Myc. Mol. Cancer.

[93] Tu W.B., Shiah Y.-J., Lourenco C., Mullen P.J., Dingar D., Redel C., Tamachi A., Ba-Alawi W., Aman A., Al-awar R. (2018). MYC Interacts with the G9a Histone Methyltransferase to Drive Transcriptional Repression and Tumorigenesis. Cancer Cell.

[94] Mabe N.W., Garcia N.M.G., Wolery S.E., Newcomb R., Meingasner R.C., Vilona B.A., Lupo R., Lin C.-C., Chi J.-T., Alvarez J.V. (2020). G9a Promotes Breast Cancer Recurrence through Repression of a Pro-Inflammatory Program. Cell Rep..

[95] Kim T.W., Cheon C., Ko S.-G. (2020). SH003 Activates Autophagic Cell Death by Activating ATF4 and Inhibiting G9a under Hypoxia in Gastric Cancer Cells. Cell Death Dis..

[96] Chang C.-C., Wu M.-J., Yang J.-Y., Camarillo I.G., Chang C.-J. (2015). Leptin–STAT3–G9a Signaling Promotes Obesity-Mediated Breast Cancer Progression. Cancer Res..

[97] Choi Y.K., Cho S.-G., Woo S.-M., Yun Y.J., Park S., Shin Y.C., Ko S.-G. (2014). Herbal Extract SH003 Suppresses Tumor Growth and Metastasis of MDA-MB-231 Breast Cancer Cells by Inhibiting STAT3-IL-6 Signaling. Mediat. Inflamm..

[98] Song H., Wang R., Wang S., Lin J. (2005). A Low-Molecular-Weight Compound Discovered through Virtual Database Screening Inhibits Stat3 Function in Breast Cancer Cells. Proc. Natl. Acad. Sci. USA.

[99] Dong J., Cheng X.-D., Zhang W.-D., Qin J.-J. (2021). Recent Update on Development of Small-Molecule STAT3 Inhibitors for Cancer Therapy: From Phosphorylation Inhibition to Protein Degradation. J. Med. Chem..

[100] Zhao M., Jiang B., Gao F.-H. (2011). Small Molecule Inhibitors of STAT3 for Cancer Therapy. Curr. Med. Chem..

[101] Chae Y.-C., Kim J.-Y., Park J.W., Kim K.-B., Oh H., Lee K.-H., Seo S.-B. (2019). FOXO1 Degradation via G9a-Mediated Methylation Promotes Cell Proliferation in Colon Cancer. Nucleic Acids Res..

[102] Koga H. (2003). Troglitazone Induces P27Kip1-Associated Cell-Cycle Arrest through down-Regulating Skp2 in Human Hepatoma Cells. Hepatology.

[103] Hsu J.-D., Kao S.-H., Ou T.-T., Chen Y.-J., Li Y.-J., Wang C.-J. (2011). Gallic Acid Induces G2/M Phase Arrest of Breast Cancer Cell MCF-7 through Stabilization of P27 ^Kip1^ Attributed to Disruption of P27 ^Kip1^/Skp2 Complex. J. Agric. Food Chem..

[104] Ungermannova D., Lee J., Zhang G., Dallmann H.G., McHenry C.S., Liu X. (2013). High-Throughput Screening AlphaScreen Assay for Identification of Small-Molecule Inhibitors of Ubiquitin E3 Ligase SCFSkp2-Cks1. J. Biomol. Screen..

[105] Singh R., Sran A., Carroll D.C., Huang J., Tsvetkov L., Zhou X., Sheung J., McLaughlin J., Issakani S.D., Payan D.G. (2015). Developing Structure–Activity Relationships from an HTS Hit for Inhibition of the Cks1–Skp2 Protein–Protein Interaction. Bioorg. Med. Chem. Lett..

[106] Ooi L.-C., Watanabe N., Futamura Y., Sulaiman S.F., Darah I., Osada H. (2013). Identification of Small Molecule Inhibitors of P27Kip1 Ubiquitination by High-Throughput Screening. Cancer Sci..

[107] Lee S.H., Hyeon D.Y., Yoon S.-H., Jeong J.-H., Han S.-M., Jang J.-W., Nguyen M.P., Chi X.-Z., An S., Hyun K. (2021). RUNX3 Methylation Drives Hypoxia-Induced Cell Proliferation and Antiapoptosis in Early Tumorigenesis. Cell Death Differ..

[108] Purcell D.J., Khalid O., Ou C.-Y., Little G.H., Frenkel B., Baniwal S.K., Stallcup M.R. (2012). Recruitment of Coregulator G9a by Runx2 for Selective Enhancement or Suppression of Transcription. J. Cell. Biochem..

[109] Liu X., Miao Z., Wang Z., Zhao T., Xu Y., Song Y., Huang J., Zhang J., Xu H., Wu J. (2018). TBX2 Overexpression Promotes Proliferation and Invasion through Epithelial-mesenchymal Transition and ERK Signaling Pathway. Exp. Ther. Med..

[110] Dutta A., Le Magnen C., Mitrofanova A., Ouyang X., Califano A., Abate-Shen C. (2016). Identification of an NKX3.1-G9a-UTY Transcriptional Regulatory Network That Controls Prostate Differentiation. Science.

[111] Dong C., Wu Y., Yao J., Wang Y., Yu Y., Rychahou P.G., Evers B.M., Zhou B.P. (2012). G9a Interacts with Snail and Is Critical for Snail-Mediated E-Cadherin Repression in Human Breast Cancer. J. Clin. Investig..

[112] Yingling J.M., McMillen W.T., Yan L., Huang H., Sawyer J.S., Graff J., Clawson D.K., Britt K.S., Anderson B.D., Beight D.W. (2018). Preclinical Assessment of Galunisertib (LY2157299 Monohydrate), a First-in-Class Transforming Growth Factor-β Receptor Type I Inhibitor. Oncotarget.

[113] Kim B.-G., Malek E., Choi S.H., Ignatz-Hoover J.J., Driscoll J.J. (2021). Novel Therapies Emerging in Oncology to Target the TGF-β Pathway. J. Hematol. Oncol..

[114] Hau P., Jachimczak P., Schlingensiepen R., Schulmeyer F., Jauch T., Steinbrecher A., Brawanski A., Proescholdt M., Schlaier J., Buchroithner J. (2007). Inhibition of TGF-Beta2 with AP 12009 in Recurrent Malignant Gliomas: From Preclinical to Phase I/II Studies. Oligonucleotides.

[115] Hu Y., Zheng Y., Dai M., Wang X., Wu J., Yu B., Zhang H., Cui Y., Kong W., Wu H. (2019). G9a and Histone Deacetylases Are Crucial for Snail2-mediated E-cadherin Repression and Metastasis in Hepatocellular Carcinoma. Cancer Sci..

[116] Pfeiffer N., Voykov B., Renieri G., Bell K., Richter P., Weigel M., Thieme H., Wilhelm B., Lorenz K., Feindor M. (2017). First-in-Human Phase I Study of ISTH0036, an Antisense Oligonucleotide Selectively Targeting Transforming Growth Factor Beta 2 (TGF-Β2), in Subjects with Open-Angle Glaucoma Undergoing Glaucoma Filtration Surgery. PLoS ONE.

[117] Gimeno-Valiente F., Riffo-Campos Á.L., Torres L., Tarazona N., Gambardella V., Cervantes A., López-Rodas G., Franco L., Castillo J. (2021). Epigenetic Mechanisms Are Involved in the Oncogenic Properties of ZNF518B in Colorectal Cancer. Cancers.

[118] Li Z., Jiao X., Di Sante G., Ertel A., Casimiro M.C., Wang M., Katiyar S., Ju X., Klopfenstein D.V., Tozeren A. (2019). Cyclin D1 Integrates G9a-Mediated Histone Methylation. Oncogene.

[119] Yang Q., Zhu Q., Lu X., Du Y., Cao L., Shen C., Hou T., Li M., Li Z., Liu C. (2017). G9a Coordinates with the RPA Complex to Promote DNA Damage Repair and Cell Survival. Proc. Natl. Acad. Sci. USA.

[120] Si M., Lang J. (2018). The Roles of Metallothioneins in Carcinogenesis. J. Hematol. Oncol..

[121] Curry E., Green I., Chapman-Rothe N., Shamsaei E., Kandil S., Cherblanc F.L., Payne L., Bell E., Ganesh T., Srimongkolpithak N. (2015). Dual EZH2 and EHMT2 Histone Methyltransferase Inhibition Increases Biological Efficacy in Breast Cancer Cells. Clin. Epigenetics.

[122] Ishiguro K., Kitajima H., Niinuma T., Maruyama R., Nishiyama N., Ohtani H., Sudo G., Toyota M., Sasaki H., Yamamoto E. (2021). Dual EZH2 and G9a Inhibition Suppresses Multiple Myeloma Cell Proliferation by Regulating the Interferon Signal and IRF4-MYC Axis. Cell Death Discov..

[123] Alao J.P., Lam E.W.-F., Ali S., Buluwela L., Bordogna W., Lockey P., Varshochi R., Stavropoulou A.V., Coombes R.C., Vigushin D.M. (2004). Histone Deacetylase Inhibitor Trichostatin a Represses Estrogen Receptor α-Dependent Transcription and Promotes Proteasomal Degradation of Cyclin D1 in Human Breast Carcinoma Cell Lines. Clin. Cancer Res..

[124] San José-Enériz E., Agirre X., Rabal O., Vilas-Zornoza A., Sanchez-Arias J.A., Miranda E., Ugarte A., Roa S., Paiva B., Estella-Hermoso de Mendoza A. (2017). Discovery of First-in-Class Reversible Dual Small Molecule Inhibitors against G9a and DNMTs in Hematological Malignancies. Nat. Commun..

[125] Liu Y., Liu S., Yuan S., Yu H., Zhang Y., Yang X., Xie G., Chen Z., Li W., Xu B. (2017). Chromodomain Protein CDYL Is Required for Transmission/Restoration of Repressive Histone Marks. J. Mol. Cell Biol..

[126] Wu H., Zhang H., Wang P., Mao Z., Feng L., Wang Y., Liu C., Xia Q., Li B., Zhao H. (2013). Short-Form CDYLb but Not Long-Form CDYLa Functions Cooperatively with Histone Methyltransferase G9a in Hepatocellular Carcinomas. Genes Chromosomes Cancer.

[127] Yang L., Liu Y., Fan M., Zhu G., Jin H., Liang J., Liu Z., Huang Z., Zhang L. (2019). Identification and Characterization of Benzo[d]Oxazol-2(3H)-One Derivatives as the First Potent and Selective Small-Molecule Inhibitors of Chromodomain Protein CDYL. Eur. J. Med. Chem..

[128] Wang S., Wu G., Han Y., Song P., Chen J., Wu Y., Yang J., Liang P. (2018). MiR-124 Regulates STAT3-mediated Cell Proliferation, Migration and Apoptosis in Bladder Cancer. Oncol. Lett..

[129] Song W., Gu Y., Lu S., Wu H., Cheng Z., Hu J., Qian Y., Zheng Y., Fan H. (2019). LncRNA TRERNA1 Facilitates Hepatocellular Carcinoma Metastasis by Dimethylating H3K9 in the CDH1 Promoter Region via the Recruitment of the EHMT2/SNAI1 Complex. Cell Prolif..

[130] Li Y., Cheng C. (2018). Long Noncoding RNA NEAT1 Promotes the Metastasis of Osteosarcoma via Interaction with the G9a-DNMT1-Snail Complex. Am. J. Cancer Res..

[131] Li Q., Dong C., Cui J., Wang Y., Hong X. (2018). Over-Expressed LncRNA HOTAIRM1 Promotes Tumor Growth and Invasion through up-Regulating HOXA1 and Sequestering G9a/EZH2/Dnmts Away from the HOXA1 Gene in Glioblastoma Multiforme. J. Exp. Clin. Cancer Res. CR.

[132] West L.E., Gozani O. (2011). Regulation of P53 Function by Lysine Methylation. Epigenomics.

[133] Huang J., Dorsey J., Chuikov S., Zhang X., Jenuwein T., Reinberg D., Berger S.L. (2010). G9a and Glp Methylate Lysine 373 in the Tumor Suppressor P53. J. Biol. Chem..

[134] Nakatsuka T., Tateishi K., Kato H., Fujiwara H., Yamamoto K., Kudo Y., Nakagawa H., Tanaka Y., Ijichi H., Ikenoue T. (2021). Inhibition of Histone Methyltransferase G9a Attenuates Liver Cancer Initiation by Sensitizing DNA-Damaged Hepatocytes to P53-Induced Apoptosis. Cell Death Dis..

[135] Dang C.V. (1999). C-Myc Target Genes Involved in Cell Growth, Apoptosis, and Metabolism. Mol. Cell. Biol..

[136] Ke X.X., Zhang R., Zhong X., Zhang L., Cui H. (2020). Deficiency of G9a Inhibits Cell Proliferation and Activates Autophagy via Transcriptionally Regulating C-Myc Expression in Glioblastoma. Front. Cell Dev. Biol..

[137] Siveen K.S., Sikka S., Surana R., Dai X., Zhang J., Kumar A.P., Tan B.K.H., Sethi G., Bishayee A. (2014). Targeting the STAT3 Signaling Pathway in Cancer: Role of Synthetic and Natural Inhibitors. Biochim. Biophys. Acta Rev. Cancer.

[138] Ivanov V.N., Bhoumik A., Krasilnikov M., Raz R., Owen-Schaub L.B., Levy D., Horvath C.M., Ronai Z. (2001). Cooperation between STAT3 and C-Jun Suppresses Fas Transcription. Mol. Cell.

[139] Tzivion G., Dobson M., Ramakrishnan G. (2011). FoxO Transcription Factors; Regulation by AKT and 14-3-3 Proteins. Biochim. Biophys. Acta BBA Mol. Cell Res..

[140] Van der Heide L.P., Hoekman M.F.M., Smidt M.P. (2004). The Ins and Outs of FoxO Shuttling: Mechanisms of FoxO Translocation and Transcriptional Regulation. Biochem. J..

[141] Chen F., Liu X., Bai J., Pei D., Zheng J. (2016). The Emerging Role of RUNX3 in Cancer Metastasis (Review). Oncol. Rep..

[142] Crawford N.T., McIntyre A.J., McCormick A., D’Costa Z.C., Buckley N.E., Mullan P.B. (2019). TBX2 Interacts with Heterochromatin Protein 1 to Recruit a Novel Repression Complex to EGR1-Targeted Promoters to Drive the Proliferation of Breast Cancer Cells. Oncogene.

[143] Zhu B., Zhang M., Williams E.M., Keller C., Mansoor A., Davie J.K. (2016). TBX2 Represses PTEN in Rhabdomyosarcoma and Skeletal Muscle. Oncogene.

[144] Zhu L.-F., Hu Y., Yang C.-C., Xu X.-H., Ning T.-Y., Wang Z.-L., Ye J.-H., Liu L.-K. (2012). Snail Overexpression Induces an Epithelial to Mesenchymal Transition and Cancer Stem Cell-like Properties in SCC9 Cells. Lab. Investig..

[145] Zhou W., Lv R., Qi W., Wu D., Xu Y., Liu W., Mou Y., Wang L. (2014). Snail Contributes to the Maintenance of Stem Cell-Like Phenotype Cells in Human Pancreatic Cancer. PLoS ONE.

[146] Wang Y., Shi J., Chai K., Ying X., Zhou B.P. (2013). The Role of Snail in EMT and Tumorigenesis. Curr. Cancer Drug Targets.

[147] Dong C., Yuan T., Wu Y., Wang Y., Fan T.W.M., Miriyala S., Lin Y., Yao J., Shi J., Kang T. (2013). Loss of FBP1 by Snail-Mediated Repression Provides Metabolic Advantages in Basal-like Breast Cancer. Cancer Cell.

[148] Walsh H.R., Cruickshank B.M., Brown J.M., Marcato P. (2019). The Flick of a Switch: Conferring Survival Advantage to Breast Cancer Stem Cells Through Metabolic Plasticity. Front. Oncol..

[149] Medici D., Hay E.D., Olsen B.R. (2008). Snail and Slug Promote Epithelial-Mesenchymal Transition through ␤-Catenin–T-Cell Factor-4-Dependent Expression of Transforming Growth Factor-␤3. Mol. Biol. Cell.

[150] Hu Y., Zheng Y., Dai M., Wu J., Yu B., Zhang H., Kong W., Wu H., Yu X. (2019). Snail2 Induced E-Cadherin Suppression and Metastasis in Lung Carcinoma Facilitated by G9a and HDACs. Cell Adhes. Migr..

[151] Bian C., Chen Q., Yu X. (2015). The Zinc Finger Proteins ZNF644 and WIZ Regulate the G9a/GLP Complex for Gene Repression. eLife.

[152] Mayer D., Stadler M.B., Rittirsch M., Hess D., Lukonin I., Winzi M., Smith A., Buchholz F., Betschinger J. (2020). Zfp281 Orchestrates Interconversion of Pluripotent States by Engaging Ehmt1 and Zic2. EMBO J..

[153] Topacio B.R., Zatulovskiy E., Cristea S., Xie S., Tambo C.S., Rubin S.M., Sage J., Kõivomägi M., Skotheim J.M. (2019). Cyclin D-Cdk4,6 Drives Cell-Cycle Progression via the Retinoblastoma Protein’s C-Terminal Helix. Mol. Cell.

[154] Massagué J. (2008). TGFβ in Cancer. Cell.

[155] Van Steensel B., Belmont A.S. (2017). Lamina-Associated Domains: Links with Chromosome Architecture, Heterochromatin and Gene Repression. Cell.

[156] Li L., Guan Y., Chen X., Yang J., Cheng Y. (2021). DNA Repair Pathways in Cancer Therapy and Resistance. Front. Pharmacol..

[157] Zou Y., Liu Y., Wu X., Shell S.M. (2006). Functions of Human Replication Protein A (RPA): From DNA Replication to DNA Damage and Stress Responses. J. Cell. Physiol..

[158] Han Y.-C., Zheng Z.-L., Zuo Z.-H., Yu Y.P., Chen R., Tseng G.C., Nelson J.B., Luo J.-H. (2013). Metallothionein 1 h Tumour Suppressor Activity in Prostate Cancer Is Mediated by Euchromatin Methyltransferase 1: MT1h Suppresses Prostate Cancer through Activation of EHMT1. J. Pathol..

[159] Zheng Y., Jiang L., Hu Y., Xiao C., Xu N., Zhou J., Zhou X. (2017). Metallothionein 1H (MT1H) Functions as a Tumor Suppressor in Hepatocellular Carcinoma through Regulating Wnt/β-Catenin Signaling Pathway. BMC Cancer.

[160] Mozzetta C., Pontis J., Fritsch L., Robin P., Portoso M., Proux C., Margueron R., Ait-Si-Ali S. (2014). The Histone H3 Lysine 9 Methyltransferases G9a and GLP Regulate Polycomb Repressive Complex 2-Mediated Gene Silencing. Mol. Cell.

[161] Fritsch L., Robin P., Mathieu J.R.R., Souidi M., Hinaux H., Rougeulle C., Harel-Bellan A., Ameyar-Zazoua M., Ait-Si-Ali S. (2010). A Subset of the Histone H3 Lysine 9 Methyltransferases Suv39h1, G9a, GLP, and SETDB1 Participate in a Multimeric Complex. Mol. Cell.

[162] Estève P.-O., Chin H.G., Smallwood A., Feehery G.R., Gangisetty O., Karpf A.R., Carey M.F., Pradhan S. (2006). Direct Interaction between DNMT1 and G9a Coordinates DNA and Histone Methylation during Replication. Genes Dev..

[163] Boulias K. (2004). Functional Role of G9a-Induced Histone Methylation in Small Heterodimer Partner-Mediated Transcriptional Repression. Nucleic Acids Res..

[164] Mulligan P., Westbrook T.F., Ottinger M., Pavlova N., Chang B., Macia E., Shi Y.-J., Barretina J., Liu J., Howley P.M. (2008). CDYL Bridges REST and Histone Methyltransferases for Gene Repression and Suppression of Cellular Transformation. Mol. Cell.

[165] Caron C., Pivot-Pajot C., Van Grunsven L.A., Col E., Lestrat C., Rousseaux S., Khochbin S. (2003). Cdyl: A New Transcriptional Co-repressor. EMBO Rep..

[166] Qiu Z., Zhu W., Meng H., Tong L., Li X., Luo P., Yi L., Zhang X., Guo L., Wei T. (2019). CDYL Promotes the Chemoresistance of Small Cell Lung Cancer by Regulating H3K27 Trimethylation at the CDKN1C Promoter. Theranostics.

[167] Siouda M., Dujardin A.D., Barbollat-Boutrand L., Mendoza-Parra M.A., Gibert B., Ouzounova M., Bouaoud J., Tonon L., Robert M., Foy J.-P. (2020). CDYL2 Epigenetically Regulates MIR124 to Control NF-ΚB/STAT3-Dependent Breast Cancer Cell Plasticity. iScience.

[168] Jeong D., Kim J., Nam J., Sun H., Lee Y.-H., Lee T.-J., Aguiar R.C.T., Kim S.-W. (2015). MicroRNA-124 Links P53 to the NF-ΚB Pathway in B-Cell Lymphomas. Leukemia.

[169] Ribeiro D.M., Zanzoni A., Cipriano A., Delli Ponti R., Spinelli L., Ballarino M., Bozzoni I., Tartaglia G.G., Brun C. (2018). Protein Complex Scaffolding Predicted as a Prevalent Function of Long Non-Coding RNAs. Nucleic Acids Res..

[170] Morriss G.R., Cooper T.A. (2017). Protein Sequestration as a Normal Function of Long Noncoding RNAs and a Pathogenic Mechanism of RNAs Containing Nucleotide Repeat Expansions. Hum. Genet..

[171] O’Driscoll M., Jeggo P.A. (2006). The Role of Double-Strand Break Repair—Insights from Human Genetics. Nat. Rev. Genet..

[172] Uddin F., Rudin C.M., Sen T. (2020). CRISPR Gene Therapy: Applications, Limitations, and Implications for the Future. Front. Oncol..

[173] Yang Y., Xu J., Ge S., Lai L. (2021). CRISPR/Cas: Advances, Limitations, and Applications for Precision Cancer Research. Front. Med..

[174] Saraon P., Pathmanathan S., Snider J., Lyakisheva A., Wong V., Stagljar I. (2021). Receptor Tyrosine Kinases and Cancer: Oncogenic Mechanisms and Therapeutic Approaches. Oncogene.

[175] Normanno N., De Luca A., Bianco C., Strizzi L., Mancino M., Maiello M.R., Carotenuto A., De Feo G., Caponigro F., Salomon D.S. (2006). Epidermal Growth Factor Receptor (EGFR) Signaling in Cancer. Gene.

[176] Parag-Sharma K., Tasoulas J., Musicant A.M., Nascimento-Filho C.H.V.D., Zhu Z., Twomey C., Liu P., Castilho R.M., Amelio A.L. (2021). Synergistic Efficacy of Combined EGFR and HDAC Inhibitors Overcomes Tolerance to EGFR Monotherapy in Salivary Mucoepidermoid Carcinoma. Oral Oncol..

[177] Deutsch A.J.A., Angerer H., Fuchs T.E., Neumeister P. (2012). The Nuclear Orphan Receptors NR4A as Therapeutic Target in Cancer Therapy. Anticancer Agents Med. Chem..

[178] Song J., Diao F., Ma X., Xu S., Cui Y., Jiang S., Liu J. (2019). Androgen Upregulates NR4A1 via the TFAP2A and ETS Signaling Networks. Int. J. Biochem. Cell Biol..

[179] Yang X., Sun L., Wang L., Yao B., Mo H., Yang W. (2019). LncRNA SNHG7 Accelerates the Proliferation, Migration and Invasion of Hepatocellular Carcinoma Cells via Regulating MiR-122-5p and RPL4. Biomed. Pharmacother..

[180] Dey S.K., Jaffrey S.R. (2019). RIBOTACs: Small Molecules Target RNA for Degradation. Cell Chem. Biol..

[181] Hirota K. (2021). HIF-α Prolyl Hydroxylase Inhibitors and Their Implications for Biomedicine: A Comprehensive Review. Biomedicines.

[182] Lu H., Zhou Q., He J., Jiang Z., Peng C., Tong R., Shi J. (2020). Recent Advances in the Development of Protein–Protein Interactions Modulators: Mechanisms and Clinical Trials. Signal Transduct. Target. Ther..

[183] Yang D., Kumar S., Zhang X., Qin D., Kang S., King K., Qiu S., Liu M., Nikolovska-Coleska Z., McEachern D. (2007). Preclinical Characterization of MI-219, A Novel, Potent and Orally Active Small Molecule Inhibitor of the MDM2-P53 Interaction. Cancer Res..

[184] Whitfield J.R., Beaulieu M.-E., Soucek L. (2017). Strategies to Inhibit Myc and Their Clinical Applicability. Front. Cell Dev. Biol..

[185] Han H., Jain A.D., Truica M.I., Izquierdo-Ferrer J., Anker J.F., Lysy B., Sagar V., Luan Y., Chalmers Z.R., Unno K. (2019). Small-Molecule MYC Inhibitors Suppress Tumor Growth and Enhance Immunotherapy. Cancer Cell.

[186] Hart J.R., Garner A.L., Yu J., Ito Y., Sun M., Ueno L., Rhee J.-K., Baksh M.M., Stefan E., Hartl M. (2014). Inhibitor of MYC Identified in a Kröhnke Pyridine Library. Proc. Natl. Acad. Sci. USA.

[187] Demma M.J., Mapelli C., Sun A., Bodea S., Ruprecht B., Javaid S., Wiswell D., Muise E., Chen S., Zelina J. (2019). Omomyc Reveals New Mechanisms to Inhibit the MYC Oncogene. Mol. Cell. Biol..

[188] Ali A., Shafarin J., Unnikannan H., Al-Jabi N., Jabal R.A., Bajbouj K., Muhammad J.S., Hamad M. (2021). Co-Targeting BET Bromodomain BRD4 and RAC1 Suppresses Growth, Stemness and Tumorigenesis by Disrupting the c-MYC-G9a-FTH1axis and Downregulation of HDAC1 in Molecular Subtypes of Breast Cancer. Int. J. Biol. Sci..

[189] Zhang H.-F., Lai R. (2014). STAT3 in Cancer—Friend or Foe?. Cancers.

[190] Seo H.-S., Ku J.M., Lee H.-J., Woo J.-K., Cheon C., Kim M., Jang B.-H., Shin Y.C., Ko S.-G. (2017). SH003 Reverses Drug Resistance by Blocking Signal Transducer and Activator of Transcription 3 (STAT3) Signaling in Breast Cancer Cells. Biosci. Rep..

[191] Cai Z., Moten A., Peng D., Hsu C.-C., Pan B.-S., Manne R., Li H., Lin H.-K. (2020). The Skp2 Pathway: A Critical Target for Cancer Therapy. Semin. Cancer Biol..

[192] Chen Q., Xie W., Kuhn D.J., Voorhees P.M., Lopez-Girona A., Mendy D., Corral L.G., Krenitsky V.P., Xu W., Moutouh-de Parseval L. (2008). Targeting the P27 E3 Ligase SCFSkp2 Results in P27- and Skp2-Mediated Cell-Cycle Arrest and Activation of Autophagy. Blood.

[193] Meng J., Ding Y., Shen A., Yan M., He F., Ji H., Zou L., Liu Y., Wang Y., Lu X. (2010). Overexpression of PPARγ Can Down-Regulate Skp2 Expression in MDA-MB-231 Breast Tumor Cells. Mol. Cell. Biochem..

[194] Chan C.-H., Morrow J.K., Li C.-F., Gao Y., Jin G., Moten A., Stagg L.J., Ladbury J.E., Cai Z., Xu D. (2013). Pharmacological Inactivation of Skp2 SCF Ubiquitin Ligase Restricts Cancer Stem Cell Traits and Cancer Progression. Cell.

[195] Pavlides S.C., Huang K.-T., Reid D.A., Wu L., Blank S.V., Mittal K., Guo L., Rothenberg E., Rueda B., Cardozo T. (2013). Inhibitors of SCF-Skp2/Cks1 E3 Ligase Block Estrogen-Induced Growth Stimulation and Degradation of Nuclear P27kip1: Therapeutic Potential for Endometrial Cancer. Endocrinology.

[196] Harney A.S., Lee J., Manus L.M., Wang P., Ballweg D.M., LaBonne C., Meade T.J. (2009). Targeted Inhibition of Snail Family Zinc Finger Transcription Factors by Oligonucleotide-Co(III) Schiff Base Conjugate. Proc. Natl. Acad. Sci. USA.

[197] Nagaraj N.S., Datta P.K. (2010). Targeting the Transforming Growth Factor-β Signaling Pathway in Human Cancer. Expert Opin. Investig. Drugs.

[198] Brandl M., Seidler B., Haller F., Adamski J., Schmid R.M., Saur D., Schneider G. (2013). IKKα Controls Canonical TGFβ–SMAD Signaling to Regulate Genes Expressing SNAIL and SLUG during EMT in Panc1 Cells. J. Cell Sci..

[199] Huang C.-Y., Chung C.-L., Hu T.-H., Chen J.-J., Liu P.-F., Chen C.-L. (2021). Recent Progress in TGF-β Inhibitors for Cancer Therapy. Biomed. Pharmacother..

[200] Morris J.C., Tan A.R., Olencki T.E., Shapiro G.I., Dezube B.J., Reiss M., Hsu F.J., Berzofsky J.A., Lawrence D.P. (2014). Phase I Study of GC1008 (Fresolimumab): A Human Anti-Transforming Growth Factor-Beta (TGFβ) Monoclonal Antibody in Patients with Advanced Malignant Melanoma or Renal Cell Carcinoma. PLoS ONE.

[201] Langenfeld J., Kiyokawa H., Sekula D., Boyle J., Dmitrovsky E. (1997). Posttranslational Regulation of Cyclin D1 by Retinoic Acid: A Chemoprevention Mechanism. Proc. Natl. Acad. Sci. USA.

[202] Mao X., Cao B., Wood T.E., Hurren R., Tong J., Wang X., Wang W., Li J., Jin Y., Sun W. (2011). A Small-Molecule Inhibitor of D-Cyclin Transactivation Displays Preclinical Efficacy in Myeloma and Leukemia via Phosphoinositide 3-Kinase Pathway. Blood.

[203] Mao X., Stewart A.K., Hurren R., Datti A., Zhu X., Zhu Y., Shi C., Lee K., Tiedemann R., Eberhard Y. (2007). A Chemical Biology Screen Identifies Glucocorticoids That Regulate C-Maf Expression by Increasing Its Proteasomal Degradation through up-Regulation of Ubiquitin. Blood.

[204] Hurt E.M., Wiestner A., Rosenwald A., Shaffer A.L., Campo E., Grogan T., Bergsagel P.L., Kuehl W.M., Staudt L.M. (2004). Overexpression of C-Maf Is a Frequent Oncogenic Event in Multiple Myeloma That Promotes Proliferation and Pathological Interactions with Bone Marrow Stroma. Cancer Cell.

[205] Tiedemann R.E., Mao X., Shi C.-X., Zhu Y.X., Palmer S.E., Sebag M., Marler R., Chesi M., Fonseca R., Bergsagel P.L. (2008). Identification of Kinetin Riboside as a Repressor of CCND1 and CCND2 with Preclinical Antimyeloma Activity. J. Clin. Investig..

